# Molecular characterization of arenavirus defective viral genomes reveals sequence features associated with their formation

**DOI:** 10.1128/jvi.01978-25

**Published:** 2025-12-09

**Authors:** Thomas Hoenen, Patrick Bohn, Sebastian Herndler, Marine-Noël Klamke, Andreas Müller, Allison Groseth

**Affiliations:** 1 Laboratory for Integrative Cell and Infection Biology, Institute of Molecular Virology and Cell Biology, Friedrich-Loeffler-Institut 39023, Greifswald, Germany; 2 Laboratory for Arenavirus Biology, Institute of Molecular Virology and Cell Biology, Friedrich-Loeffler-Institut 39023, Greifswald, Germany; University Medical Center Freiburg, Freiburg, Germany

**Keywords:** arenavirus, Tacaribe virus, defective viral genomes, MinION, nanopore sequencing

## Abstract

Defective viral genomes (DVGs) are byproducts of replication that arise during infection with diverse RNA viruses and can impact virus infection and disease outcome. To gain insight into DVG generation during arenavirus infection, we serially passaged Tacaribe virus at a high multiplicity of infection, which led to the generation of both deletion DVGs (del-DVGs) and copyback DVGs (cb-DVGs). Interestingly, specific combinations of start/stop breakpoints were highly overrepresented, resulting in certain DVGs being highly enriched within the population. Functional characterization of the most prevalent del-DVGs suggests that they are able to compete for interaction with the viral RNA synthesis machinery and that this ability is length-dependent. A closer analysis of the DVG breakpoints used to generate highly abundant DVGs revealed a role for local sequence identity in the formation of cb-DVGs, while del-DVG formation was associated with the presence of specific nucleotide triplets (i.e., TAG, AGA, and GAA). Taken together with similar findings from other virus families, this then supports the idea that DVG formation is not a random process, but rather that specific mechanisms promote their formation at certain positions. The characterization of these individual arenavirus DVG sequences, and also the identification of sequence elements associated with their production, will facilitate future work examining their impact on arenavirus biology, and also opens up the possibility of using such sequences as a part of antiviral approaches and/or of modulating their production as a part of virus attenuation strategies.

**IMPORTANCE:**

Infection with diverse RNA viruses can generate defective viral genomes (DVGs) that, while unable to support productive virus infection on their own, appear to play a crucial role in determining infection outcome. In light of this apparent biological importance, there is an urgent need to better understand the sequence characteristics of individual DVGs and the molecular mechanisms that regulate their formation to study their biological functions. We have now characterized several DVGs that are highly enriched during infection with the arenavirus Tacaribe virus. Functional analysis of a subset of these DVGs showed length-dependent competition for the viral RNA synthesis machinery, while detailed sequence analysis revealed that DVG formation involves either regions of sequence identity within the genome or the presence of specific nucleotide sequences. Understanding these mechanisms opens up the possibility to leverage DVG generation in support of antiviral and/or vaccine attenuation approaches.

## INTRODUCTION

Members of the family *Arenaviridae* that infect mammals (i.e., members of the genus *Mammarenavirus*) are maintained in persistently infected hosts, primarily rodents, from which they can be transmitted to humans, where many of them then represent important human pathogens. These include Old World arenaviruses such as lymphocytic choriomeningitis virus (LCMV), which causes aseptic meningitis, encephalitis, and/or meningoencephalitis (1), as well as Lassa virus (LASV) and Lujo virus, both of which are causative agents of hemorrhagic fever in Africa (2, 3). In addition, there are a number of arenaviruses endemic to South America (New World arenaviruses), for example, Junín virus (JUNV) and Machupo virus (MACV), that are highly pathogenic in humans and cause severe hemorrhagic fevers (4, 5). However, this group also contains a number of closely related viruses, including Tacaribe virus (TCRV), that are not pathogenic in humans and are, therefore, often used to study the basic biology of these viruses under BSL2 conditions and for comparative studies to identify traits that play a role in pathogenesis.

**Fig 1:**
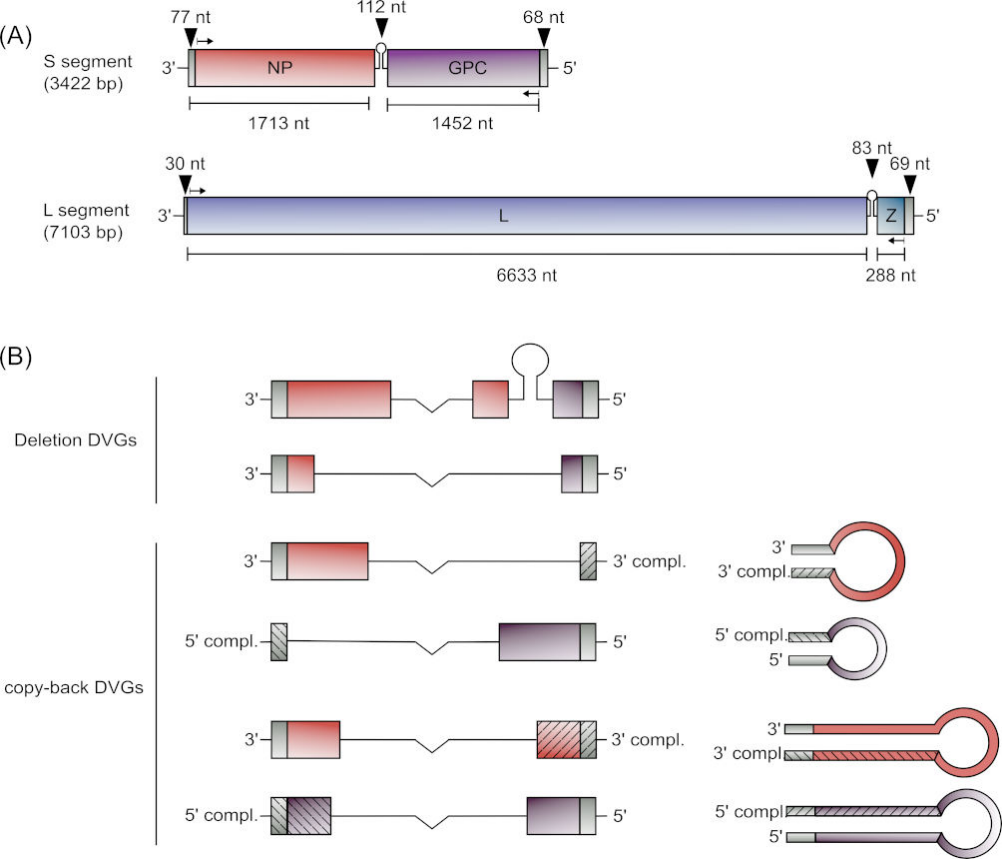
Schematic of the arenavirus genome and different defective viral genome (DVG) types. (**A**) TCRV genome structure. The TCRV genome is composed of two ambisense ssRNA genome segments: a large (L) segment, with a length of 7.1 kb, and a small S segment, with a length of 3.4 kb. The S segment encodes the nucleoprotein (NP) and the glycoprotein precursor (GPC), while the L segment encodes the viral polymerase (L) and matrix protein (Z). Both segments contain noncoding regions in the form of the 3′ and 5′ terminal UTRs and the IGR separating the two open reading frames. (**B**) DVG structures. Different possible DVG structures are shown using the S segment as an example. Deletion DVGs contain internal deletions in their coding regions but retain intact 3′ and 5′ UTRs. In contrast, copyback DVGs contain complementary terminal regions that can either be restricted to the non-coding UTRs or can include coding regions as well, and thus they can form regions of dsRNA with varying length.

Mammalian arenaviruses have a bi-segmented, ambisense single-stranded RNA (ssRNA) genome (6) with the S segment (ca. 3.4 kb) encoding the viral nucleoprotein (NP) and the glycoprotein precursor (GPC), while the L segment (ca. 7.1 kb) encodes the RNA-dependent RNA polymerase (L) and the matrix protein (Z) (Fig. 1A). Both genome segments contain a highly structured, noncoding intergenic region (IGR) that separates the two encoded ORFs and acts as a transcription termination signal during mRNA transcription (7). Additionally, both segments include untranslated regions (UTRs) at the 3′ and 5′ genome ends that are critically necessary for replication, as they contain the genomic and antigenomic promoters, respectively (8).

In addition to standard (full length) viral genomes, RNA viruses are capable of generating defective viral genomes (DVGs) as a by-product of their replication (reviewed in reference 9). In general, there are two broad types of DVGs that have been described: deletion DVGs (del-DVGs) and copyback DVGs (cb-DVGs). del-DVGs retain both the natural 3′ and 5′ UTRs of the parental genome but contain deletions of internal portions of the genome (Fig. 1B) that can range from a few nucleotides to almost the entire genome (reviewed in reference 10). They have been shown to occur during infection with a wide range of both positive- and negative-sense RNA viruses, including those with segmented genomes, such as influenza virus (reviewed in references 11, 12). In contrast, cb-DVGs contain a portion of the genome flanked by either the 3′ or 5′ UTR, and its reverse complement (Fig. 1B). Such cb-DVGs can be generated during the replication of the genome (vRNA) or antigenome (cRNA), and correspondingly result in either 3′ UTR cb-DVGs (i.e., containing the 3′ UTR and its reverse complement, generated during replication of vRNA) or 5′ UTR cb-DVGs (i.e., comprising the 5′ UTR and its reverse complement, produced during replication of cRNA). The formation of cb-DVGs has been observed during infection with many different non-segmented negative-sense RNA viruses (i.e., members of the *Paramyxoviridae*, *Pneumoviridae*, *Rhabdoviridae,* and *Filoviridae* families) (reviewed in reference 12).

The existence of DVGs has been known for many decades, but only recently have we seen a resurgence in interest regarding their biological relevance, which has revealed a number of important roles in virus biology and disease outcome. This can occur through (i) competition with standard virus genomes for cellular and viral resources needed for virus replication, (ii) by serving as ligands for pathways associated with antiviral immunity, and (iii) by facilitating virus persistence (reviewed in references 13, 14). In particular, cb-DVGs can have a significant degree of dsRNA character and thus have the potential to be recognized by dsRNA sensors, such as those associated with interferon production (reviewed in reference 15). Consistent with the increasing recognition of their biological importance, there is also tremendous interest in identifying specific mechanism(s) that may regulate DVG formation. Intriguingly, for both influenza virus (16) and respiratory syncytial virus (RSV) (17), studies have shown that DVG formation occurs at certain genome locations where specific nucleotide sequences are present, indicating that DVG generation is not entirely random, but rather involves specific mechanisms that regulate this process to at least some extent.

For arenaviruses, subgenomic RNA production in the form of DVGs has long been known to occur both *in vitro* and *in vivo* (18–22), and indeed their generation and packaging into defective interfering (DI) particles is postulated to play an important role in arenavirus biology, and especially in persistence (18–23). The earliest detailed information on the sequences of such DVGs revealed the generation of unusual DVG forms that had either lost or gained nucleotides in the genome termini during persistent LCMV infection of cell cultures (19). However, more recent studies based on next-generation sequencing have now started to also reveal the formation of classical del-DVGs, including an LCMV del-DVG with a deletion in the IGR and the end of the GPC gene that appears to exert interfering activity by limiting GPC expression (24). Importantly, arenaviruses have also recently been shown to possess an unusual dsRNA-specific exonuclease activity encoded by the viral NP that has been suggested to degrade viral dsRNAs that could otherwise serve as pathogen-associated molecular patterns (PAMPs) for the activation of diverse RNA sensors (25–31). While the origins and structural characteristics of such viral dsRNA ligands remain unclear, one potential source of these structures could be cb-DVGs. Indeed, recent work has also shown the formation of cb-DVGs for LCMV, although interestingly, they were observed to only an extremely limited extent for New World arenaviruses (24). This is surprising since dsRNA accumulation during infection with New World arenaviruses has been reported to be much more extensive than with Old World arenaviruses (32) and suggests that further work characterizing these structures remains needed.

To gain insights into the sequence characteristics of individual DVGs being generated during arenavirus infection, as well as the mechanism(s) that underlie their generation, we repeatedly passaged the arenavirus TCRV at a high multiplicity of infection (MOI) to induce the formation and accumulation of DVGs, which were then sequenced using nanopore technology and analyzed using a set of scripts that we developed for the detection of both del-DVGs and cb-DVGs in these kinds of long single-read sequencing data sets.

## RESULTS

### Accumulation of aberrant small RNAs during serial passaging of TCRV

**Fig 2:**
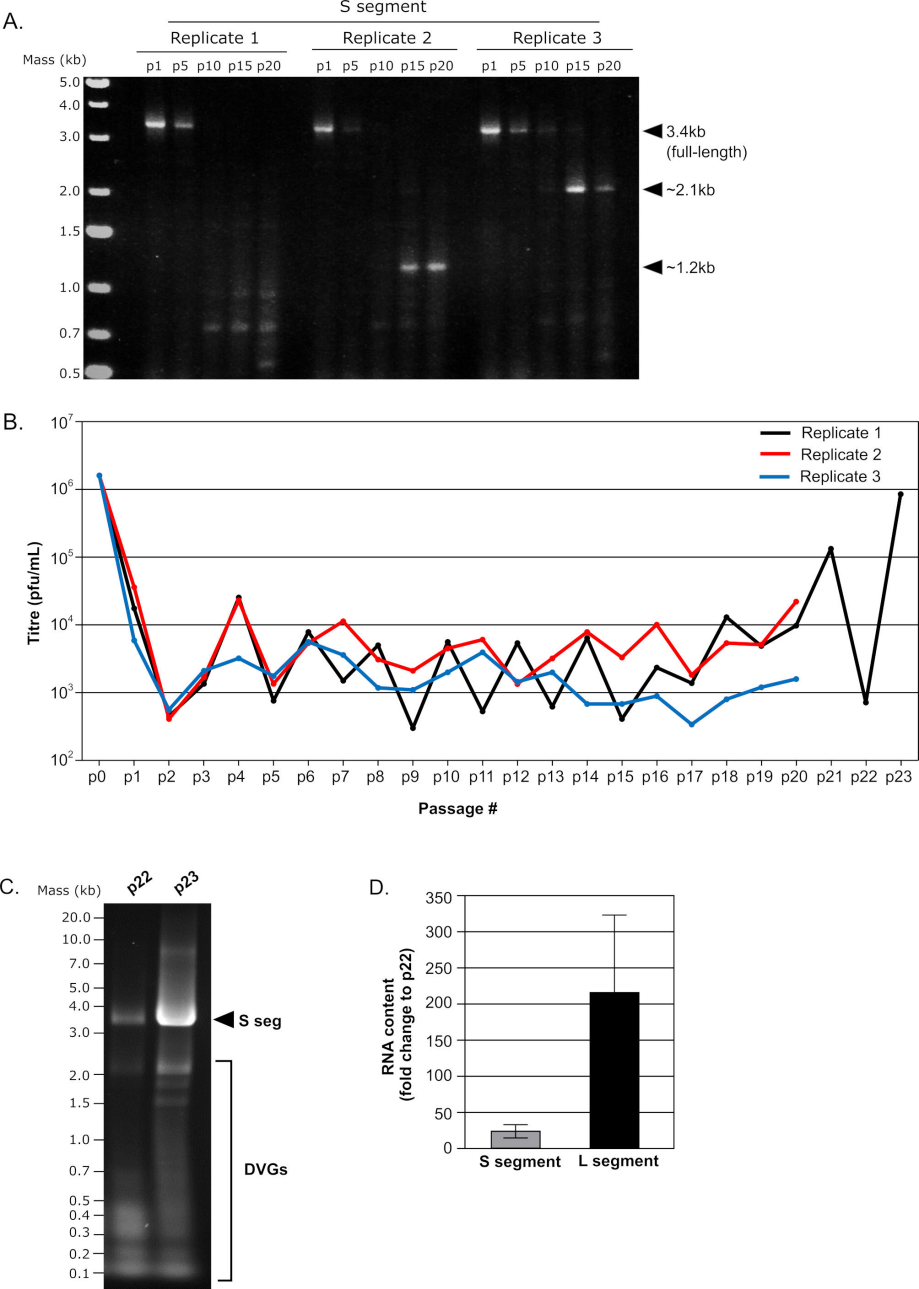
Serial passaging of TCRV stocks in Vero cells. (**A**) Accumulation of TCRV DVGs. RNA of three independently generated replicates of TCRV serially passaged in Vero 76 cells was isolated from cell supernatant after 1 (p1), 5 (p5), 10 (p10), 15 (p15), or 20 (p20) passages, as indicated. The obtained RNA was then reverse transcribed with a universal TCRV genome end primer and amplified by PCR using S segment-specific genome end primers. The full-length S segment and smaller S segment-derived products are indicated with their approximate sizes. (**B**) Virus titers. Supernatants from each passage from p1 to p20 were analyzed by plaque assay for determination of viral titers. In addition, one replicate (replicate 1) was additionally passaged from p20 to p23, resulting in a spontaneous restoration of viral titers. (**C**) Comparison of genome and DVG content in p22 and p23 TCRV stocks. RNA from the p22 and p23 virus stocks was isolated and amplified as described in (**A**). The full-length S segment and smaller S segment-derived products are indicated. (**D**) Comparison of full-length genome content. RNA isolated from an equivalent number of infectious particles from either p22 or p23 was subjected to RT using a genome-end binding primer followed by qPCR amplification using primers binding to central portions of the viral genome within the NP and L open reading frames (as a proxy for full-length S and L-segment content). The fold increase in genome content between p22 and p23 stocks was then calculated and is shown as the mean and standard deviation of three replicate experiments.

To encourage the formation of DVGs, we performed serial passaging of TCRV over 20 passages in three independent replicates. RT-PCR amplification of the extracted viral RNA using primers targeting the S segment 3′ and 5′ UTRs showed evidence for the accumulation of smaller viral genome products with increasing passage number, while the abundance of full-length S segment products diminished (Fig. 2A). Examination of virus titers over the course of the passaging showed an initial 2–3 log reduction in the p1 and p2 samples, after which the titer fluctuated cyclically within a 1–1.5 log range throughout the rest of the passaging experiment (Fig. 2B). Intriguingly, further passaging of the generated p20 stock for one replicate resulted in a spontaneous 3 log increase in virus titer between p22 and p23, which was associated with a reduced DVG content and increased levels of the full-length S segment RNA (Fig. 2B and C). Estimation of full-length genome content by RT-qPCR indicated that particles within the p23 stock contained substantially more intact S (i.e., 20×) and L (200× more) segments than those from the p22 stock (Fig. 2D). While DVGs can also be generated without the need for genome rearrangement through the accumulation of changes at individual genome positions that render these viral genome copies functionally defective, Sanger sequencing of both the S and L genome segments failed to reveal the accumulation of changes that were consistently present throughout the passaging process, were present as majority populations, and/or were present consistently across replicates (Fig. S1).

**Fig 3:**
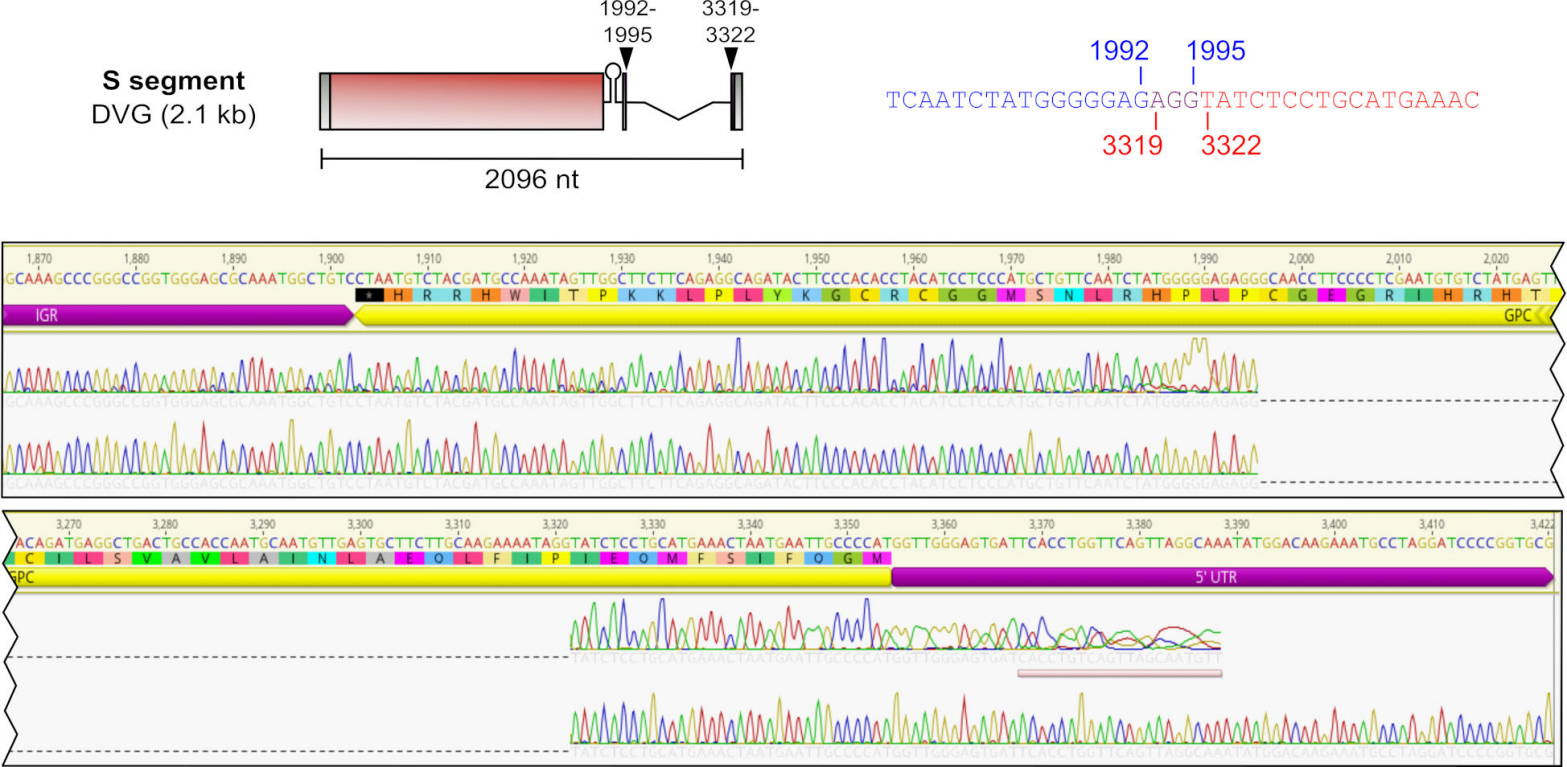
Schematic representation and sequence of the observed TCRV S segment 2.1 kb del-DVG. Both the general structure of this DVG (top left) and the details of the sequence surrounding the break point (top right) are shown. The sequence before the break starts (blue), after the break stops (red), and ambiguous nucleotides that could belong to either end (purple) are indicated along with their respective positions within the genome. The corresponding Sanger sequencing data indicate that this product represents a del-DVG in which nearly the entire GPC gene (yellow region marker) has been deleted.

Given these indications that the observed truncated TCRV DVGs were exerting interfering activity during infection, we sought to more precisely define the sequences of the individual DVGs. While efforts to Sanger sequence the 1.2 kb subgenomic product were unsuccessful, the larger 2.1 kb product produced clear Sanger sequencing data that revealed that this product was an S segment-derived del-DVG lacking the majority of the GPC gene (Fig. 3). Interestingly, it was not possible to unequivocally define the precise break start/stop for this del-DVG due to identity in the sequences before (i.e., positions 1993–1995) and after the putative breakpoint (i.e., positions 3319–3321). As such, the observed AGG found at the breakpoint in the del-DVG sequence could be formed by joining several combinations of nucleotides between positions 1992–1995 and 3319–3322 (i.e., 1992::3319, 1993::3320, 1994::3321, or 1995::3322) (Fig. 3).

**Fig 4:**
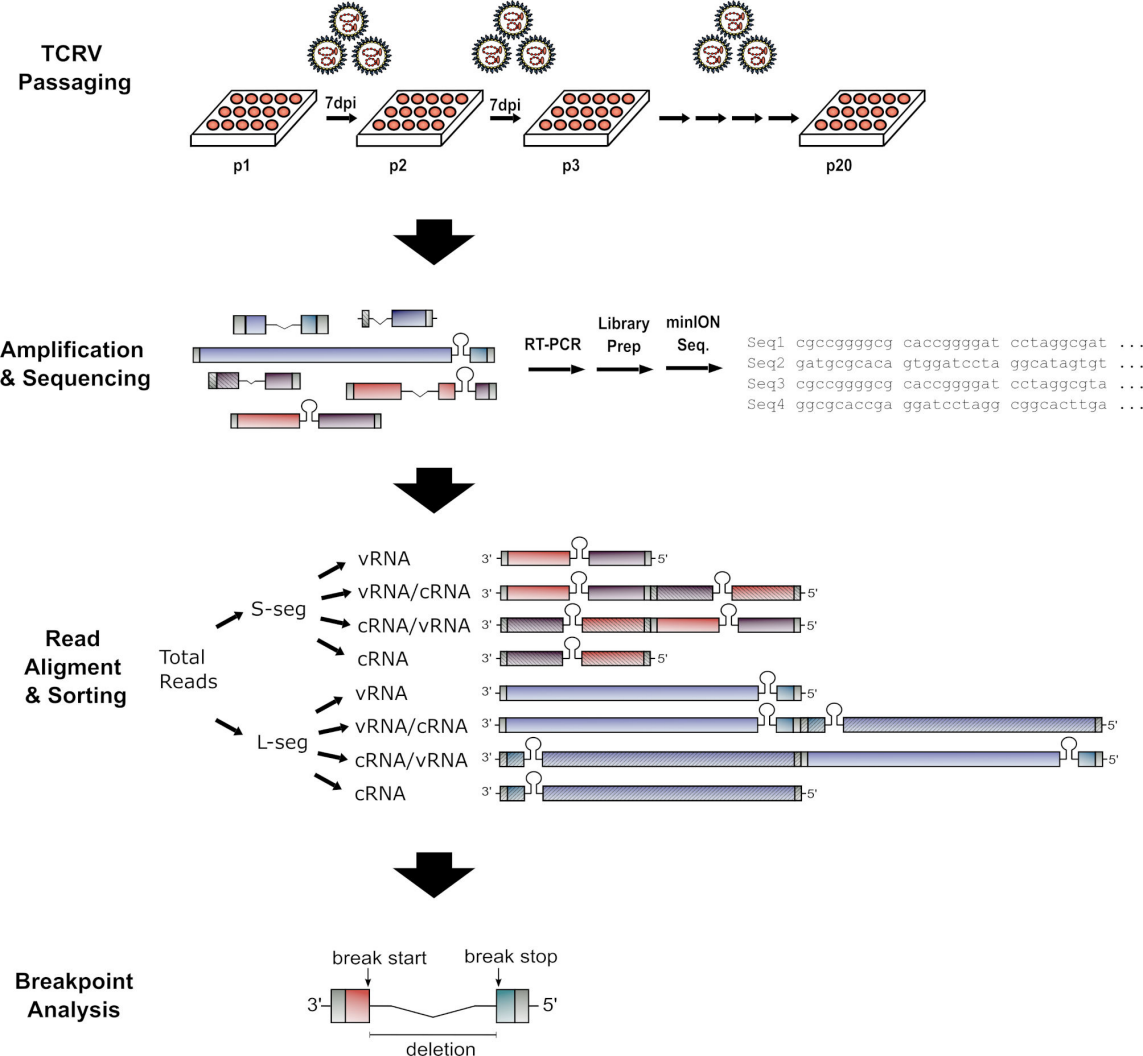
Sample analysis and data processing workflow. TCRV passaging: TCRV was subjected to serial passage in Vero76 cells to promote DVG accumulation. Amplification and sequencing: RNA was isolated from passage 20 supernatants and converted to cDNA using a universal genome end primer. This was followed by separate polymerase chain reactions using segment-specific genome end primers. The resulting products were used for library preparation using the nanopore 1D2 kit and loaded on a MinION sequencer. Read sorting: Obtained sequences were sorted using flexbar (v3.1) into S-segment (red/purple) or L-segment (blue/turquoise) sequences based on the presence of the primer sequences used for their amplification and then further subdivided based on local alignment using lastal (version 921) into those that best aligned to a (i) vRNA-sense genome, (ii) cRNA-sense genome, (iii) vRNA-cRNA concatemer, or (iv) cRNA-vRNA concatemer (schematic representations of the templates used for these local alignments are shown). Breakpoint analysis: Once sorted, reads were trimmed, and breakpoints in the DVG sequences were identified by global alignment to the appropriate reference template (i.e., vRNA, cRNA, cRNA_vRNA or cRNA_cRNA) using a high gap open penalty (i.e., 50) but a very low gap extend penalty (0.000000001) to allow for the large deletions expected in DVGs. Break start and stop points for each DVG were then extracted for further analysis.

In addition to these two larger products, a number of smaller products of different sizes, and in varying abundance, were also observed (Fig. 2). To gain greater insight into the genetic structure of these putative DVGs, a long single-read sequencing approach was deemed necessary. The workflow for this approach is summarized in Fig. 4, with the resulting PCR products (Fig. S2) being pooled and subjected to nanopore sequencing on a MinION device. This analysis resulted in 652,000 sequencing reads, with 581,781 reads found to be specific for TCRV, based on the presence of sequences corresponding to the genome termini (Fig. S3A). Of these, 395,736 reads corresponded to the S segment, and 186,045 reads corresponded to the L segment.

### Identification and classification of DVG reads

Analysis of reads mapping to the S and L segments with gaps >100 nt led to 307,079 (S segment) and 165,614 (L segment) reads being preliminarily identified as putative DVGs. A more detailed examination of these reads revealed that a small number (0.4% of reads) corresponded to concatemers of short internal del-DVGs, most likely representing a PCR artifact, so that these sequences were discarded from further analysis. Thus, our analysis ultimately resulted in 306,537 sequences for S-segment DVGs and 162,297 sequences for L-segment DVGs, which were then used for further analysis (Fig. S3A). More detailed examination of the identified DVG reads showed products that resembled not only vRNA or cRNA (i.e., del-DVGs) but also cb-DVGs that best aligned to either a vRNA-cRNA (i.e., 3′ UTR cb-DVGs) or cRNA-vRNA (i.e., 5′ UTR cb-DVGs) concatemer reference sequence. However, these species were found with different prevalence between the S and L genome segments. For the S segment, a roughly equal proportion of reads corresponded to del-DVGs (61.0%; see Table S1 at https://zenodo.org/records/14900940) and cb-DVGs (39.0%) (Fig. S3B), while 3′ UTR cb-DVGs (38.4%; see Table S2 at https://zenodo.org/records/14900940) were clearly favored over 5′ UTR cb-DVGs (0.6%; see Table S3 at https://zenodo.org/records/14900940). In contrast, for the L segment, del-DVGs were markedly more abundant (97.2%; see Table S4 at https://zenodo.org/records/14900940) than those corresponding to cb-DVGs (2.8%) (Fig. S3B), which in this case included nearly equal proportions of 3′ UTR cb-DVGs (1.5%; see Table S5 at https://zenodo.org/records/14900940) and 5′ UTR cb-DVGs (1.3%; see Table S6 at https://zenodo.org/records/14900940).

### Breakpoint analysis: del-DVGs

**Fig 5:**
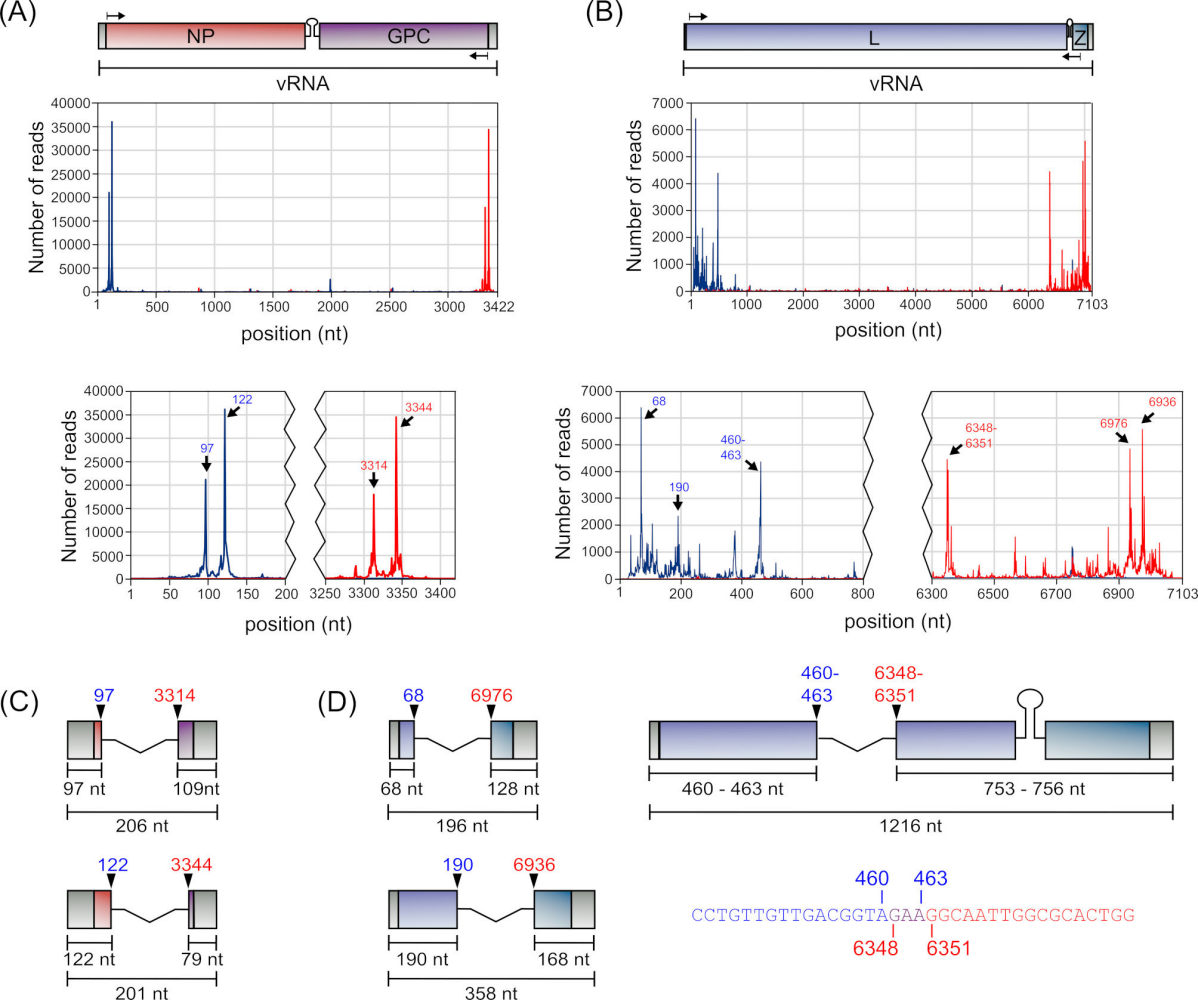
Identification of the most prevalent del-DVGs. The frequency with which del-DVG reads show break starts (blue) or break stops (red) at any given position was plotted for the (**A**) S segment or (**B**) L segment. Enlargements of the terminal regions are shown below each of the graphs. Schematic representations of highly abundant (**C**) S segment-derived or (**D**) L segment-derived del-DVGs are also shown and are drawn to scale. For DVGs with breakpoints at 460–463 and 6348–6351, which produce identical products, ambiguity in the assignment of a subset of the nucleotides (purple) to the sequence either before the break start (blue) or after the break stop (red) is indicated.

Initial breakpoint analysis for DVGs sequences involved the determination of the frequency with which individual start and stop positions were used. Among del-DVGs, a clear preference could be observed for breakpoints to form in close proximity to the genome ends, that is, within the first/last 150 bp of the S segment (Fig. 5A; see Table S7 at https://zenodo.org/records/14900940) or the first/last 500–800 bp of the L segment (Fig. 5B; see Table S8 at https://zenodo.org/records/14900940). However, the frequency with which specific breakpoints were used within these regions was not a direct function of distance from the genome termini, but rather specific positions appeared to be preferentially used.

This was particularly evident for the S segment, where two break starts were heavily favored, that is, positions 97 and 122 (Fig. 5A; see Table S7 at https://zenodo.org/records/14900940), which were collectively found 424 times more often than expected for a random distribution across the genome. These break starts, in turn, paired with heavily favored break stops at positions 3314 and 3344, resulting in DVGs with lengths of 206 nt and 201 nt, respectively (Fig. 5A and C). Alone, these two del-DVGs made up 21% of all S segment del-DVGs with single break points, and when allowing for ±1 nt accuracy, this increased to 34% (see Table S9 at https://zenodo.org/records/14900940).

While there was somewhat more variability in breakpoint positions for del-DVGs derived from the L segment, also here distinct break starts were clearly preferred, in particular at positions 68, 190, and 460–463 (Fig. 5B; see Table S8 at https://zenodo.org/records/14900940), which were found 112 times more often than expected for a random distribution across the genome. These positions also showed preferential pairing with specific break stops, that is, position 68 with 6976, position 190 with 6936, and position 460 with 6348 or position 463 with 6351 (Fig. 5B and D). Notably, however, the DVGs produced using breakpoints 460::6348 or 463::6351 correspond to identical DVG sequences (Fig. 5D), again reflecting ambiguity as to whether part of the sequence is derived from one end of the genome or the other (as we had seen with the longer 2.1 kb S segment DVG, cf. Fig. 3). As a result, our data indicate just three distinct highly prevalent L segment-derived del-DVG species, with lengths of 196, 358, and 1,216 nt, respectively (Fig. 5D). Collectively, these three DVG species make up 8.5% of the total DVG population, which increased to 9.2% when allowing for ±1 nt variation (see Table S10 at https://zenodo.org/records/14900940).

### Breakpoint analysis: S segment-derived cb-DVGs

**Fig 6:**
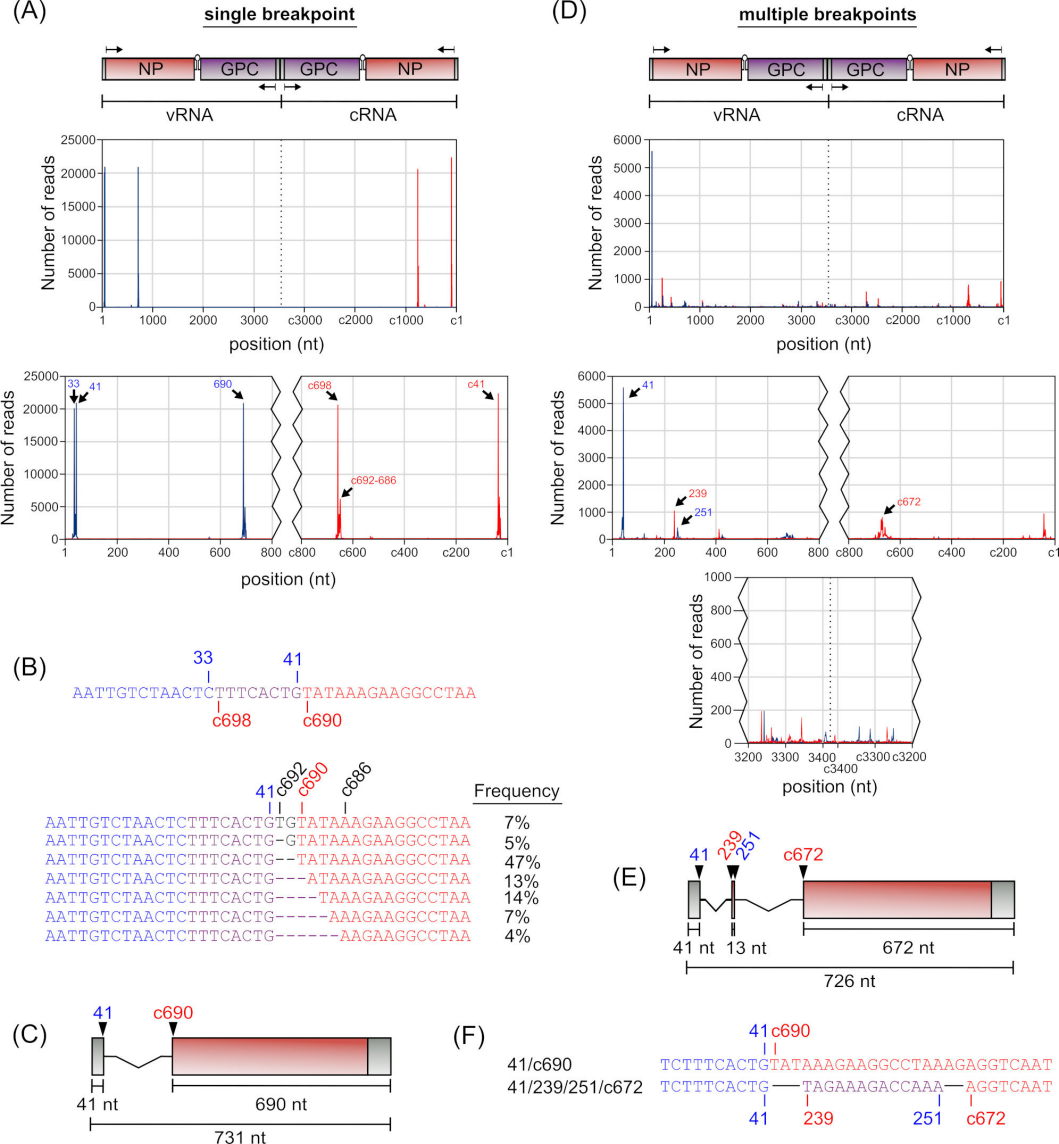
Identification of the most prevalent S segment 3′ UTR cb-DVGs. (**A**) The frequency with which cb-DVG reads with a single breakpoint show break starts (blue) or break stops (red) at any given position was plotted. Enlargements of the terminal regions are shown below the graph. (**B**) For DVGs with breakpoints at 33::c698 or 41::c690, which produce identical products, ambiguity in the assignment of a subset of the nucleotides (purple) to the sequence, either before the break start (blue) or after the break stop (red) is indicated. A detailed alignment indicating the frequency of break stop usage in the region surrounding c690 for DVGs with a break start at position 41 is also shown. (**C**) A schematic representation of the sole highly abundant S segment-derived 3′ UTR cb-DVG with a single breakpoint is shown drawn to scale. (**D**) The frequency with which cb-DVG reads with multiple breakpoints show break starts (blue) or break stops (red) at any given position was plotted. Enlargements of the terminal regions and IGR are shown below the graphs. (**E**) A schematic representation of the sole highly abundant 3′ UTR S segment-derived cb-DVG with multiple breakpoints is shown drawn to scale. (**F**) A detailed alignment comparing the sequences of DVGs produced from a single set of breakpoints at positions 41::c690, or multiple breakpoints at 41::239 and 251::c672, shows regions before the first break start (blue), between the first break stop and second break start (purple, if applicable), and after the final break stop (red).

Detailed analysis of cb-DVGs revealed two distinct populations—those with a single break start/stop (see Table S11 at https://zenodo.org/records/14900940) and those with multiple break start/stops (see Table S12 at https://zenodo.org/records/14900940). For S segment-derived 3′ UTR cb-DVGs (i.e., starting with the NP gene), we found that the vast majority (88%) of the reads contained a single breakpoint, and that these also exhibited a strong preference for specific break positions, as well as preferential break start/stop pairings (Fig. 6A, see Table S11 at https://zenodo.org/records/14900940). Specifically, break starts at positions 33, 41, and 690 were strongly favored and preferentially paired with break stops at positions c698 (i.e., position 698 in the complementary strand), nucleotides within a short region centered around c689 (i.e., c692–686), and c41, respectively (Fig. 6B). Interestingly, a closer examination of the regions adjacent to these breakpoints revealed that nucleotides 34–41 and c698–c690 were again identical in sequences, so that these cb-DVG populations are actually equivalent in sequence (Fig. 6B), as we had also seen with the 2.1 kb del-DVG identified by Sanger sequencing (c.f. Fig. 3) and one of the highly prevalent L segment-derived del-DVGs (c.f. Fig. 5D). However, in this case, the ambiguous region was longer than in those other DVGs, having a length of 8 nt, instead of only 3 nt. While this again makes it more difficult to determine which exact positions within this region are actually being used as the break start/stops, in this case, heterogeneity was observed in the break stops surrounding position c690, which indicates that it is position 41 that is likely being preferentially used for the generation of the observed cb-DVGs (Fig. 6B). Furthermore, since the DVGs with breakpoints at 41::c690 and those with breakpoints at 690::c41 are equivalent (since both strands are present for any given DVG following PCR amplification), in the end, these data suggest the presence of only a single highly abundant cb-DVG product of the S segment (Fig. 6C).

When analyzing the much less prevalent S segment 3′ UTR cb-DVGs with multiple breakpoints (see Table S12 at https://zenodo.org/records/14900940), 85% were found to have two distinct pairs of break starts/stops (see Table S2 at https://zenodo.org/records/14900940). Among these reads, 68% of initial break starts also occurred in the nucleotide regions from 33 to 41, with position 41 again being heavily favored and alone accounting for 42% of initial DVG break points (Fig. 6D; see Table S13 at https://zenodo.org/records/14900940). However, analysis of corresponding break stop sites did not reveal a clearly preferred site, although position 239 was favored, accounting for 18% of the associated break stops. Among DVGs containing an initial break start/stop between positions 41::239, a second break start at position 251 was then highly favored, accounting for 41% of the reads, and the majority of these (76%) then showed a second break stop at position c672 (Fig. 6D). The resulting DVG species then has a composition very similar to that observed for the highly prevalent single breakpoint DVG 41::c690 (cf. Fig. 6C and E), albeit with a slightly different length and internal sequence (Fig. 6F).

**Fig 7:**
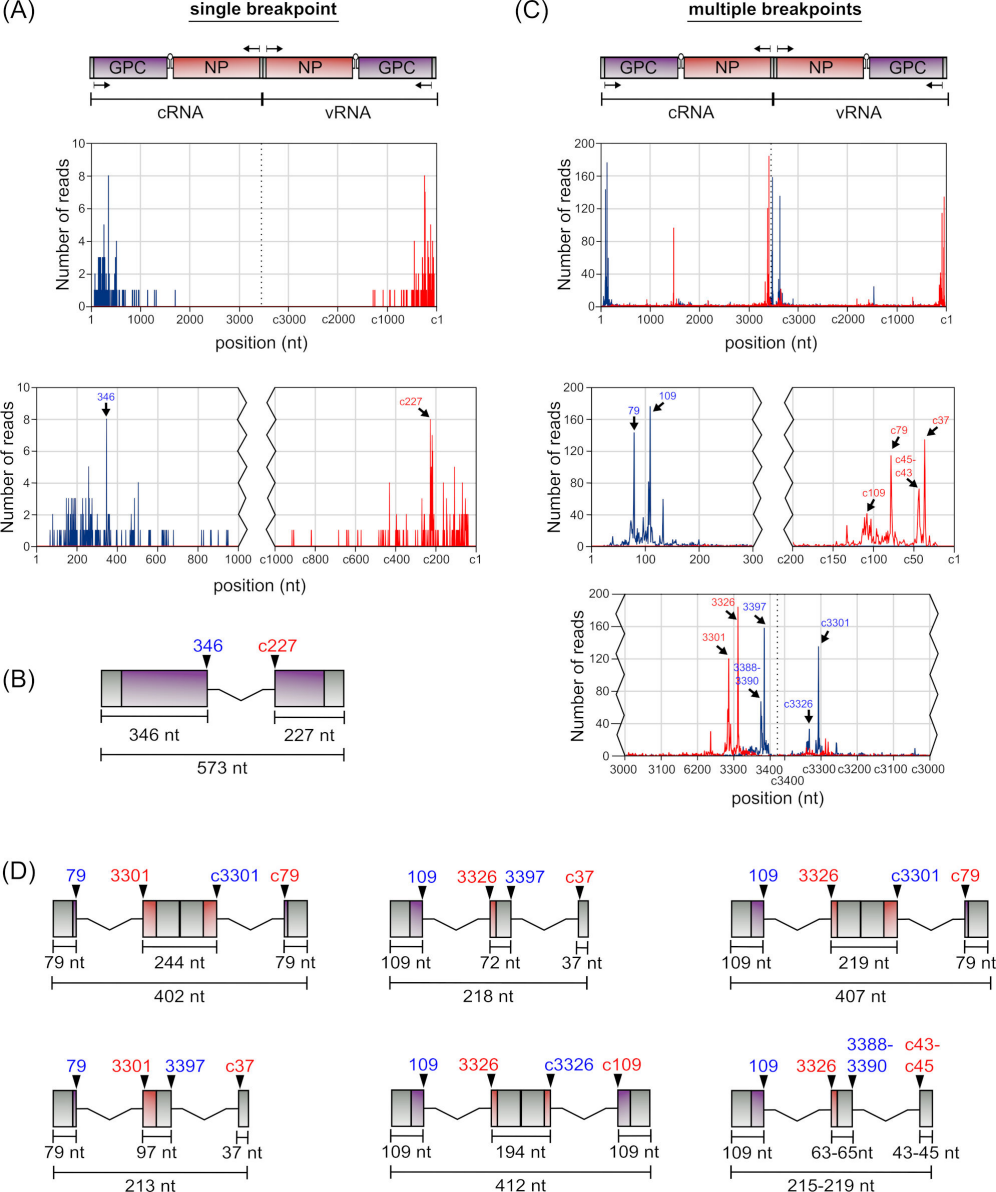
Identification of the most prevalent S segment 5′ UTR cb-DVGs. (**A**) The frequency with which cb-DVG reads with a single breakpoint show break starts (blue) or break stops (red) at any given position was plotted. Enlargements of the terminal regions are shown below the graphs. (**B**) A schematic representation of the sole highly abundant S segment-derived 5′ UTR cb-DVG with a single breakpoint is shown drawn to scale. (**C**) The frequency with which cb-DVG reads with multiple breakpoints show break starts (blue) or break stops (red) at any given position was plotted. Enlargements of the terminal regions and IGR are shown below the graphs. (**D**) Schematic representations of highly abundant S segment-derived 5′ UTR cb-DVG with multiple breakpoints are shown drawn to scale.

Analysis of S segment-derived 5′ UTR cb-DVGs (i.e., starting with the GPC gene) revealed only a small subset (10%) of reads that contained a single breakpoint (see Table S3 at https://zenodo.org/records/14900940). A detailed analysis of these reads indicated that they were highly heterogeneous, especially compared to 3′ UTR cb-DVGs for this segment (see Table S14 at https://zenodo.org/records/14900940). Reads using a break start at nucleotide 346 were the most frequently observed (5%), and always occurred together with a break stop at position c227 to produce a DVG product of 573 nt in length (Fig. 7A and B). Among DVGs containing multiple breaks (see Table S15 at https://zenodo.org/records/14900940), 51% of reads were found to contain two clear sets of break start/stops (see Table S3 at https://zenodo.org/records/14900940). Further analysis of these reads showed that two initial break points were favored, at either position 79 (14% of reads) or 109 (16% of reads) (see Table S16 at https://zenodo.org/records/14900940). Of the reads showing an initial break at position 79, the majority (71%) showed a corresponding break stop at position 3301. Of these, the largest subset (24%) then showed a second break start at position c3301, all of which then showed a second break stop at either position c79 (83%) or c78 (17%) (Fig. 7C and D, top left). Such a structure produces a perfectly complementary dsRNA of 402 nt in length that is composed almost exclusively of the viral UTRs and complementary copies thereof. A slightly smaller proportion (16%) showed initial break start/stops at 79::3301, but a second break start at position 3397 (thereby eliminating the terminal 26 nucleotides of the 3′ UTR) before reinitiating at a second break stop at position c37 (Fig. 7C and D, bottom left). As such, while this DVG exhibits complementarity between its terminal regions, there is no complement to the internal 97 nucleotides. For DVGs starting with a breakpoint at position 109, we also saw a highly preferred corresponding break stop, in this case at position 3326 (79% of reads). However, the position of the second break start was then more variable, with abundant populations using positions 3397 (12%), c3326 (11%), and c3301 (7%), as well as several related species with a break start in the range of 3388–3390 (22%) (Fig. 7D). Of the reads with a second break starting at 3397, all had a corresponding break stop at position c37 (Fig. 7D, top center), while break starts at position c3326 paired mainly with break stops at c109 (64%) (Fig. 7D, bottom center), and a further 18% pairing with a break stop at the neighboring c108. DVGs with a second break start at position c3301 paired primarily with position c79 (71%), as we had also seen for DVGs where the first break start is at position 79 (Fig. 7D, cf. top right and top left). Finally, the more variable DVG variants with a second break start in the region 3388–3390 also showed heterogeneity in their corresponding break stop positions, although those at c45–c43 were highly preferred (81%) (Fig. 7D, bottom right). In examining the resulting products, we again saw that, as for one of the cb-DVGs starting with a breakpoint at position 79 (Fig. 7D, top left), also several of these cb-DVG species starting with breaks at position 109 have the potential to form perfect (or nearly perfect) dsRNA structures over much of their length (i.e., Fig. 7D, bottom center and top right).

### Breakpoint analysis: L segment-derived cb-DVGs

**Fig 8:**
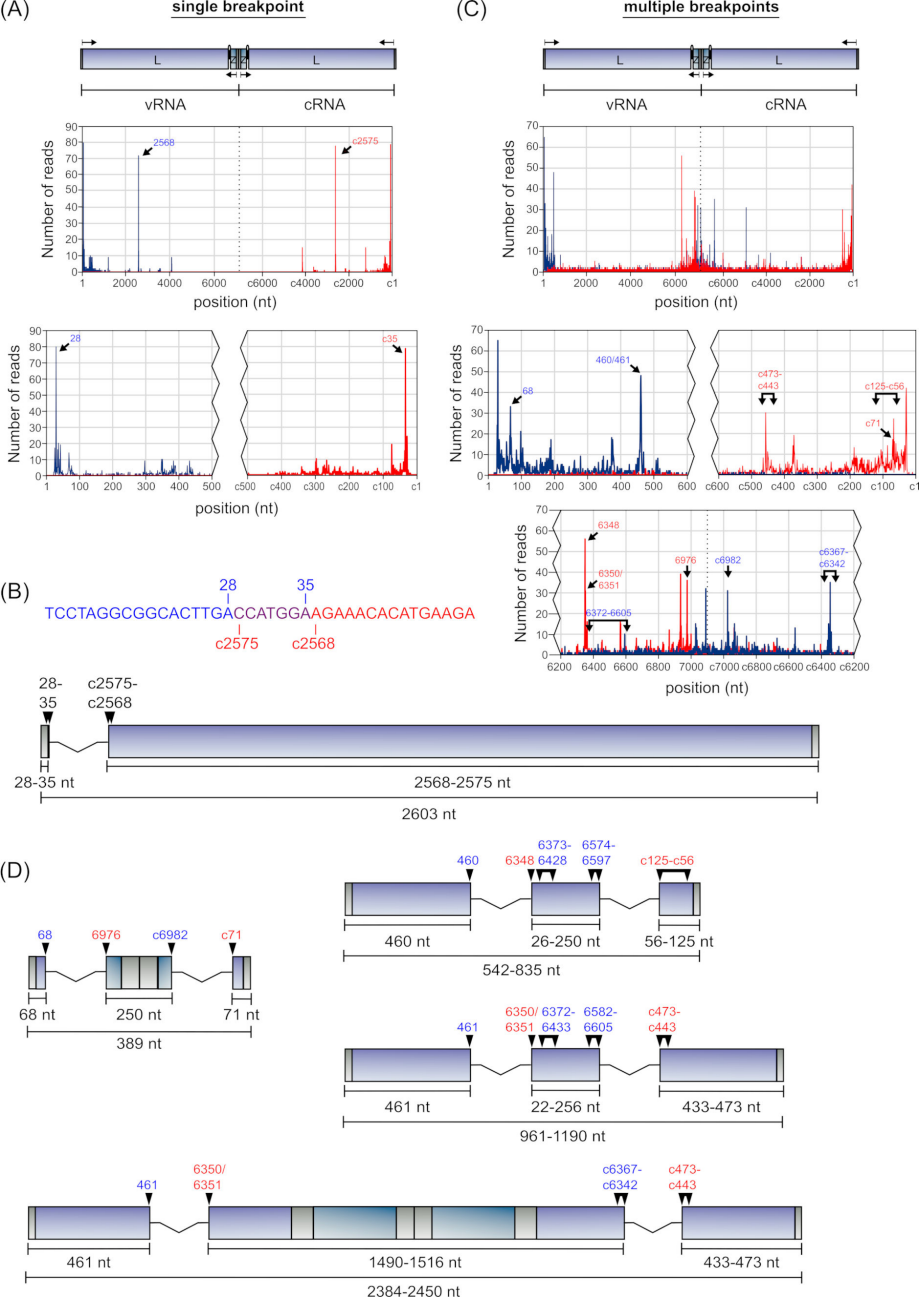
Identification of the most prevalent L segment 3′ UTR cb-DVGs. (**A**) The frequency with which cb-DVG reads with a single breakpoint show break starts (blue) or break stops (red) at any given position was plotted. Enlargements of the terminal regions are shown below the graphs. (**B**) A schematic representation of the sole highly abundant L segment-derived 3′ UTR cb-DVG with a single breakpoint is shown drawn to scale. Ambiguity in the assignment of a subset of the nucleotides (purple) to the sequence, either before the break start (blue) or after the break stop (red) is indicated. (**C**) The frequency with which cb-DVG reads with multiple breakpoints show break starts (blue) or break stops (red) at any given position was plotted. Enlargements of the terminal regions and IGR are shown below the graphs. For variable positions in which there are multiple overlapping break start-containing regions, the positions are indicated as a single combined region from 6372 to 6605. (**D**) Schematic representations of highly abundant L segment-derived 3′ UTR cb-DVG with multiple breakpoints are shown drawn to scale. For DVGs with significant variability in break point position, the longest variant is drawn, and the region within which the variable break point is found is indicated by connected arrowheads, indicating the limits of the region.

Analysis of L segment-derived 3′ UTR cb-DVGs (i.e., starting with the L gene) containing a single breakpoint, which was the case for 32% of the reads (see Table S5 at https://zenodo.org/records/14900940), also revealed two highly favored DVG populations, that is, those with break start/stops at positions 28::c2575 or 2568::c35 (see Table S17 at https://zenodo.org/records/14900940; Fig. 8A). Again, closer examination of the regions adjacent to these breakpoints showed that, as we had seen for S segment-derived 3′ UTR cb-DVGs, positions 29–35 and c2575–c2568 are identical in sequence, so that these cb-DVGs are equivalent and represent a single population where it is challenging to clearly determine which breakpoints were actually used within this region (Fig. 8B). When analyzing L segment-derived 3′ UTR cb-DVGs with multiple breakpoints (68% of the reads) (see Table S18 at https://zenodo.org/records/14900940), we found that 38% of these had two sets of break start/stops (see Table S5 at https://zenodo.org/records/14900940). More detailed analysis of these reads revealed that the most frequent break starts were positions 68, 460, and 461 (see Table S19 at https://zenodo.org/records/14900940; Fig. 8C). For DVGs starting with an initial break starting at position 68, the majority (64%) showed a break stop at position 6976, with minority populations using the nearby 6979 (14%) or 6973 (7%). Of the reads using a break stop at position 6976, 56% then had a second break start at c6982, and of those, 60% had a final break stop at position c71 (Fig. 8D, top left). In contrast, DVGs with an initial break start at 460 paired in almost all cases with 6348 (94%). The position of the second break start was variable, although it was mostly found either in the region 6373–6428 (33%) or 6574–6597 (33%). The final break stop was similarly variable, but restricted mainly to the region from c125 to c56 (70%) (Fig. 8D, top right). Finally, DVGs with an initial break start at 461, almost exclusively paired with either 6350 (76%) or 6351 (18%). Again, the second break start positions varied, but were restricted to three specific regions, that is, 6372–6433, 6582–6605, and c6367–c6342, the first two of which are very similar to those observed for DVGs starting with break positions 460::6348, supporting the propensity of specific sites within these regions to serve as a second break start. Indeed, even for the considerably longer DVGs with a second break start at c6367–c6342, this represents a very similar genome position, but within the complementary strand. The final break stop for these DVGs was also variable, but in the majority of reads (62%) was found between c473 and c433 (Fig. 8D, center right and bottom). Other commonly used breakpoints were also observed, for instance, at positions 30, 6936, 7091, and c29/c28 (see Table S18 at https://zenodo.org/records/14900940; Fig. 8C); however, these were present mainly in cb-DVGs with >2 breakpoints and were, therefore, not further analyzed in detail.

Interestingly, the multi-breakpoint 3′ UTR cb-DVGs using break starts at 68 and 460 preferentially used the same break stops we had observed with the L-segment del-DVGs, that is, 68::6976 and 460::6348 (cf. Fig. 5D and 8D). This then suggests that these DVGs might actually represent a combination of del-DVGs and cb-DVGs, although it remains unclear whether the formation of the internal deletion and the cb-DVG event would occur in parallel (i.e., during a single cycle of template replication), or whether they occur in distinct successive steps. Notably, in the case of the 3′ UTR cb-DVGs formed using a break start at position 68 (Fig. 8D, top left), and also some of those using a break start at 461 (i.e., Fig. 8D, bottom), the DVG sequences include both the 3′ and 5′ UTRs, and a complementary copy thereof. As a result, these DVGs can also form almost perfectly complementary hairpin structures containing complementary copies of both genome termini, similar to what we observed in several S segment 5′ UTR cb-DVGs (cf. Fig. 7D and 8D).

**Fig 9:**
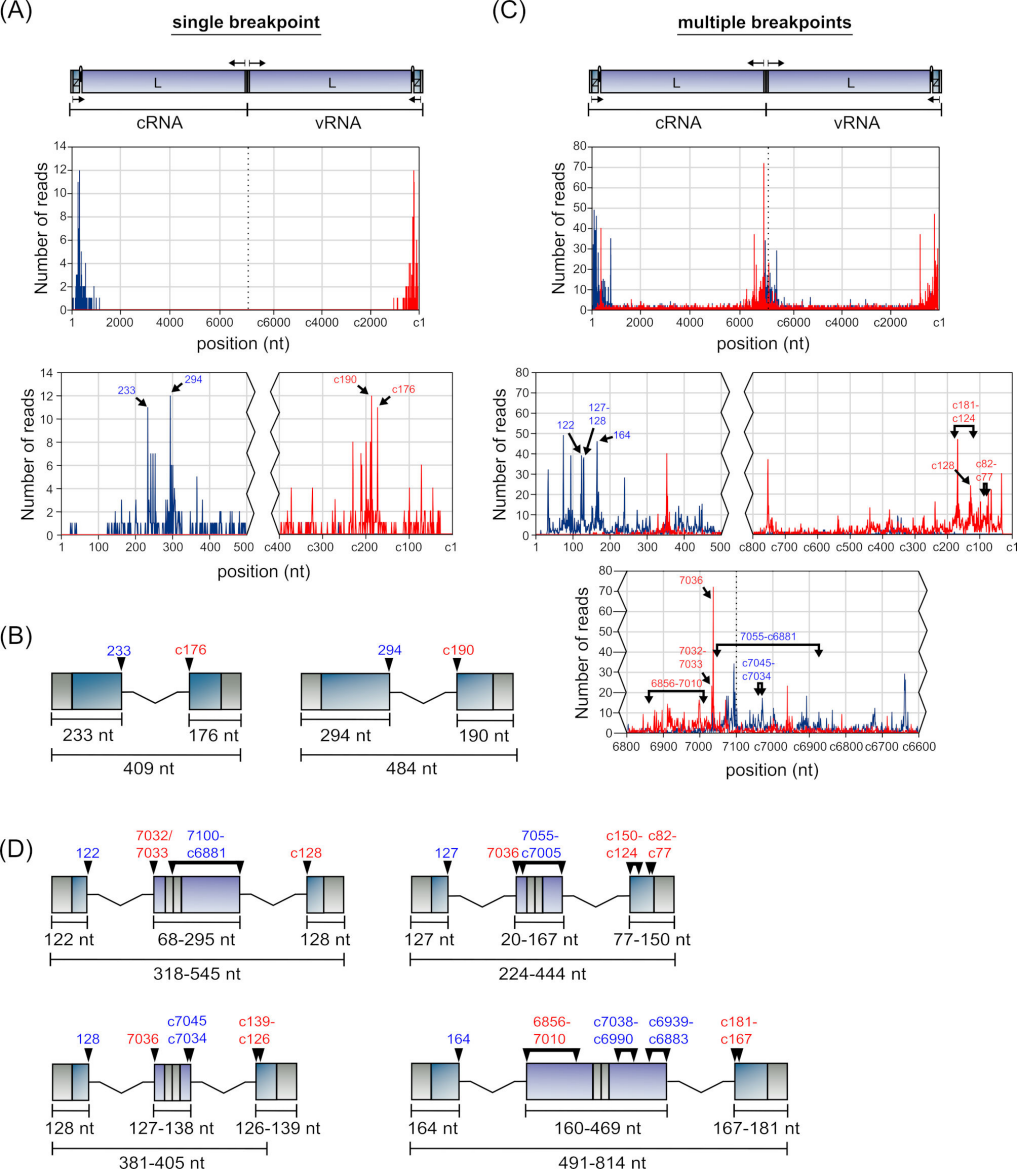
Identification of the most prevalent L segment 5′ UTR cb-DVGs. (**A**) The frequency with which cb-DVG reads with a single breakpoint show break starts (blue) or break stops (red) at any given position was plotted. Enlargements of the terminal regions are shown below the graphs. (**B**) Schematic representations of the highly abundant L segment-derived 5′ UTR cb-DVGs with a single breakpoint are shown drawn to scale. (**C**) The frequency with which cb-DVG reads with multiple breakpoints show break starts (blue) or break stops (red) at any given position was plotted. Enlargements of the terminal regions and IGR are shown below the graphs. For variable positions in which there are multiple overlapping break start/stop-containing regions, the positions are indicated as combined regions from 7055 to c6881 and c181 to c124, respectively. (**D**) Schematic representations of highly abundant L segment-derived 5′ UTR cb-DVGs with multiple breakpoints are shown drawn to scale. For DVGs with significant variability in break point position, the longest variant is drawn, and the region within which the variable break point is found is indicated by connected arrowheads, indicating the limits of the region.

When analyzing L segment-derived 5′ UTR cb-DVGs (i.e., those starting with the Z gene), we observed that reads with a single breakpoint were less frequent than what was observed for 3′ UTR cb-DVGs (i.e., starting with the L gene), and accounted for only 13% of the reads (see Table S6 at https://zenodo.org/records/14900940). Rather, this value was similar to the proportion of single breakpoint S segment-derived 5′ UTR cb-DVGs (i.e., 10%; see Table S3 at https://zenodo.org/records/14900940). Among the 5′ UTR cb-DVGs containing a single breakpoint, two break start positions were dominant, that is, 233 and 294, and these clearly paired up with c176 and c190, respectively (see Table S20 at https://zenodo.org/records/14900940; Fig. 9A and B). When analyzing cb-DVGs containing multiple breakpoints (see Table S21 at https://zenodo.org/records/14900940), which constituted 87% of the 5′ UTR cb-DVGs, 52% were found to contain two discrete pairs of break stops/starts. Also here, certain initial break start positions occurred more frequently than others, specifically 122, 127, 128, and 164 (see Table S22 at https://zenodo.org/records/14900940; Fig. 9C and D). Interestingly, while most of these clearly had preferred corresponding break stops, that is, 122::7032/7033 (81%), 127::7036 (96%), 128::7036 (65%), the break start at 164 had no clear preference for a specific break stop position. Nonetheless, there still appeared to be some restriction regarding the corresponding break stop position, given that 88% of reads with a break start at position 164 terminated within the 155 nt region from positions 6856 to 7010 (Fig. 9D, bottom right). However, for all these products, there was variability observed in the position of the second break start and stop. Specifically, the DVGs starting with 122::7032/7033 then predominantly used a second break start in the regions 7100–c6881 (76%), with the remaining break starts scattered across positions c6727–c5889, and of those, most used a second break stop at c128 (31%), with the remainder occurring primarily in the regions c140–c120 (77%) (Fig. 9D, top left). DVGs starting with 127::7036 preferentially used a second break start in the regions 7055–c7005 (55%), and those then used a second break stop mainly either within c150–c124 (50%) or c82–c77 (25%) (Fig. 9D, top right). Similarly, DVGs starting with 128::7036 preferentially used a second break start in regions c7045–c7034 (53%), with a final break stop in the regions c139–c126 (100%), with most stops occurring either at c128 or c126 (63%) (Fig. 9D, bottom left). Finally, DVGs starting with 164::6856–7010 predominantly used second break starts in the regions c7038–c6990 and c6939–c6883 (together 73%), and 73% of those then used a final break stop in the region c181–c167 (Fig. 9D, bottom right). Despite the increased variability observed with the multi-breakpoint L segment 5′ UTR cb-DVGs, in all cases the first break stops were located prior to the 3′ UTR, so that they contain complementary copies of both UTRs, and again indicating that these DVGs are the product of a combination of deletion and copyback events, similar to what we observed for the multi-breakpoint L segment 3′ UTR cb-DVGs (starting with the L gene) and the S segment 5′ UTR cb-DVGs (starting with the GPC gene).

### Functional characterization of del-DVGs

**Fig 10:**
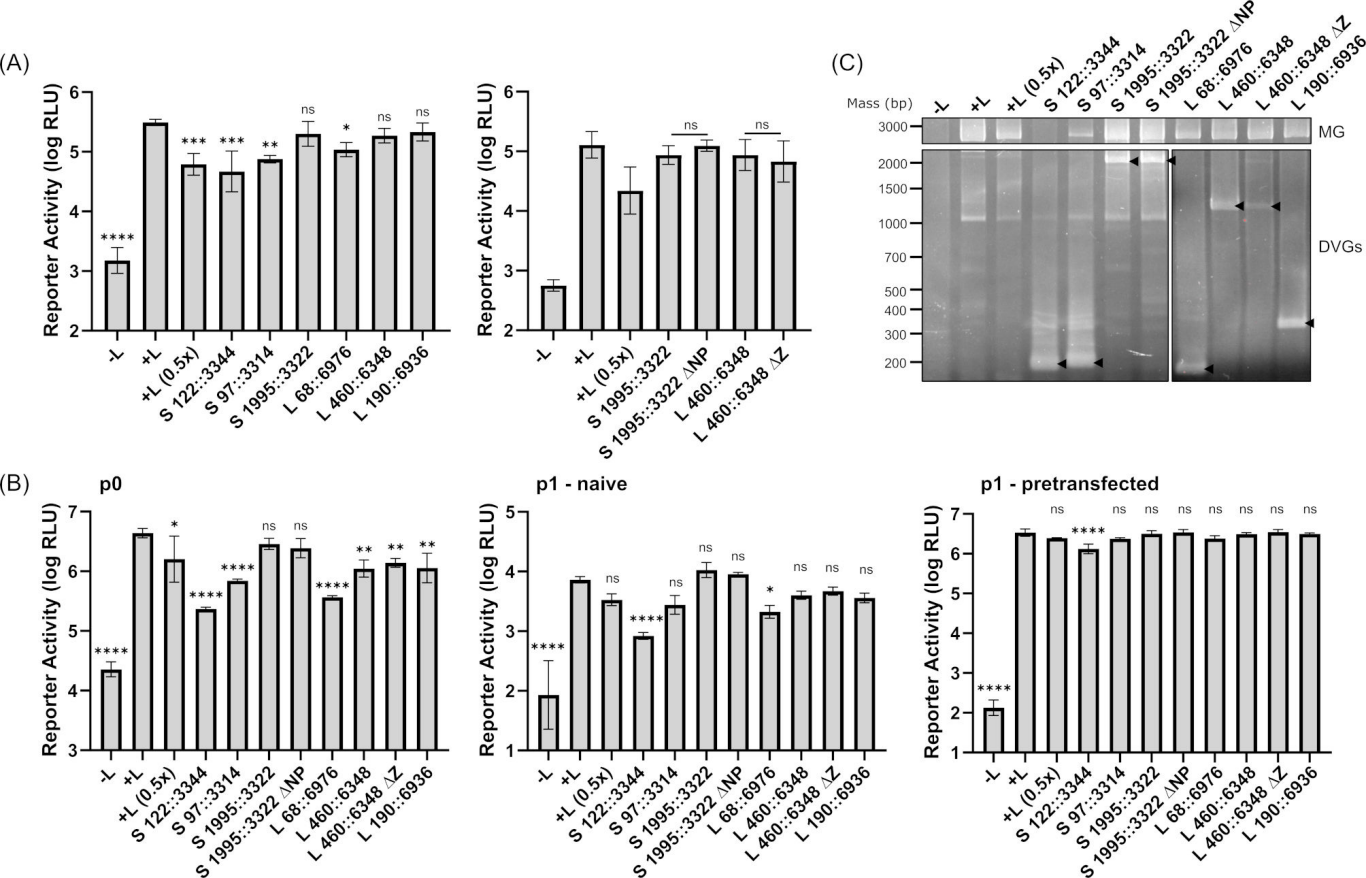
Inhibition of viral RNA synthesis by del-DVGs. (**A**) Minigenome assay. Huh7 cells were transfected with a monocistronic nanoluciferase-encoding TCRV minigenome (125 ng) and pCAGGS constructs encoding the T7 polymerase (125 ng), TCRV NP (25 ng) and polymerase (L, 100 ng), as well as pCAGGS-firefly-luciferase (FF; 50 ng) as a transfection control. Samples in which pCAGGS-L was omitted (−L) serve as a negative control, and cells transfected with only half the amount of pCAGGS-NP and pCAGGS-L serve as a control reflecting the impact of a 50% reduction in the availability of the viral NRA synthesis machinery on reporter activity. Cells were additionally transfected with the indicated del-DVG construct (125 ng), as indicated, or an equivalent amount of empty pCAGGS (+L). Cells were harvested 48 h later and measured for both nLuc (viral RNA synthesis) and FF (host cell RNA synthesis) activity. (**B**) trVLP assay. The trVLP assay was performed essentially as described in (**A**) but with transfection of a bicistronic TCRV minigenome expressing GPC-T2A-nLuc and Z (250 ng). After 72 h, these transfected (p0) cells were lysed and reporter activity measured as described in (**A**). In addition, the supernatants of these cells were collected and used to infect fresh target (P1) cells that were either untransfected (naïve) or had been transfected with pCAGGS-TCRV NP (15 ng) and pCAGGS-TCRV L (55 ng). After a further 72 h, the p1 cells were lysed, and luciferase activity was measured for nLuc (viral RNA synthesis). The means and standard deviations of three independent experiments are shown for both (**A**) and (**B**). Statistical significance was determined using one-way ANOVA (*P < 0.05, **P < 0.01, ***P < 0.001, ****P < 0.0001, ns = not significant). (**C**) Minigenome incorporation into trVLPs. RNA was isolated from supernatants of p0 cells transfected as described in (**B**) and including the indicated DVGs. The RNA was then reverse transcribed with a universal TCRV genome end primer and amplified by PCR using S segment-specific genome end primers (for minigenome, MG, and S segment-derived DVGs) or L segment-specific genome end primers (for L segment DVGs). The bicistronic minigenome (2.6 kb) is indicated in a separate panel while the positions of the individual DVGs are indicated by arrow heads (S122::3344 = 201 bp, S97::3314 = 203 bp, S1995::3322 = 2,096 bp, S::1995–3322 ∆NP = 2,096 bp, L68::6976 = 196 bp, L 460::6348 = 1,216 bp, L460::6348 ∆Z = 1,216 bp, L190::6936 = 358 bp).

To better understand the biological implications of DVG formation during arenavirus infection, we examined the impact of coexpression of each of the highly prevalent del-DVGs identified in our study (Fig. 5) in the context of both minigenome and transcription and replication-competent virus-like particle (trVLP) assays. To be able to sensitively detect competition between minigenomes and del-DVGs, the amounts of minigenome, NP, and L supplied in our assays were first titrated to establish the relevant ranges within which reporter activity is dependent on the amounts of each of these factors (Fig. S4). Based on the obtained curves, amounts of transfected minigenome (125 ng) were selected that supplied high levels of reporter activity, while amounts of NP (25 ng) and L (100 ng) were selected that were limiting. That this is the case was further demonstrated by including a control in which the amounts of NP and L were reduced by half, resulting in a strong impact on reporter activity (Fig. 10A). Similarly, reduced levels of viral RNA synthesis (as reflected by reporter activity) were observed in the minigenome assay following co-transfection of T7-driven expression constructs for several of the del-DVGs found to be highly enriched in our analysis. In particular, S122::3344 (201 bp), S97::3314 (203 bp), and L68::6976 (196 bp) all significantly reduced reporter activity in the minigenome system (Fig. 10A, left panel). Notably, these represent the shortest of the del-DVGs tested, with a length that is much shorter than that of the monocistronic minigenome (826 bp). In contrast, longer del-DVGs, that is, S1995::3322 (2,096 bp), L190::6936 (358 bp), and L460::6348 (1,216 bp) did not markedly impact the levels of reporter activity (Fig. 10A, left panel). For S1995::3322 and L460::6348, it was noted that they still contained intact open reading frames for NP and Z, respectively, and since both of these proteins contribute to the regulation of viral RNA synthesis, it was considered whether their expression might be impacting DVG function. However, we found that versions of these DVGs in which the start codons for NP or Z were eliminated behaved similarly to the parental constructs (Fig. 10A, right panel).

Since the extremely short length of the monocistronic minigenome, which is markedly shorter than the corresponding arenavirus genome segment (3.4 kb), could affect their susceptibility to competition by del-DVGs for the viral RNA synthesis machinery, the effect of del-DVG coexpression was also examined in trVLP assays, which utilize a longer bicistronic minigenome that is closer in size to the arenavirus S segment (i.e., 2.6 kb). Here, we saw that the inhibitory effects of S122::3344, S97::3314, and L68::6976 were further enhanced (Fig. 10B, left panel, p0). Furthermore, an inhibitory effect of L460::6348 and L190::6936 became apparent, although it remained weaker than for the shorter del-DVGs (Fig. 10B, left panel). This phenotype could also be transferred to fresh target (p1) cells upon infection with trVLPs generated by these DVG-expressing (p0) cells. Interestingly, however, this was only the case if the p1 target cells were naïve, that is, not pre-transfected with NP and L (Fig. 10B, center panel). Additional transfection of these components, which represent the minimal requirements for viral RNA synthesis, markedly increased the levels of reporter activity, but also masked the effects of the DVG coexpression (Fig. 10B, right panel).

Consistent with the ability of del-DVG coexpression to reduce reporter activity in target p1 cells, there was a clear reduction in the amount of incorporated minigenome in trVLPs produced in the presence of S122::3344 and S97::3314, and to a lesser extent also the L segment-derived DVGs L68::6976, L460::6348, and L190::6936 (Fig. 10C). No marked effects on minigenome incorporation were noted for the longest DVG S1995::3322, despite it also being robustly expressed and incorporated into the trVLPs (Fig. 10C).

### Identification of mechanisms contributing to DVG formation

**Fig 11:**
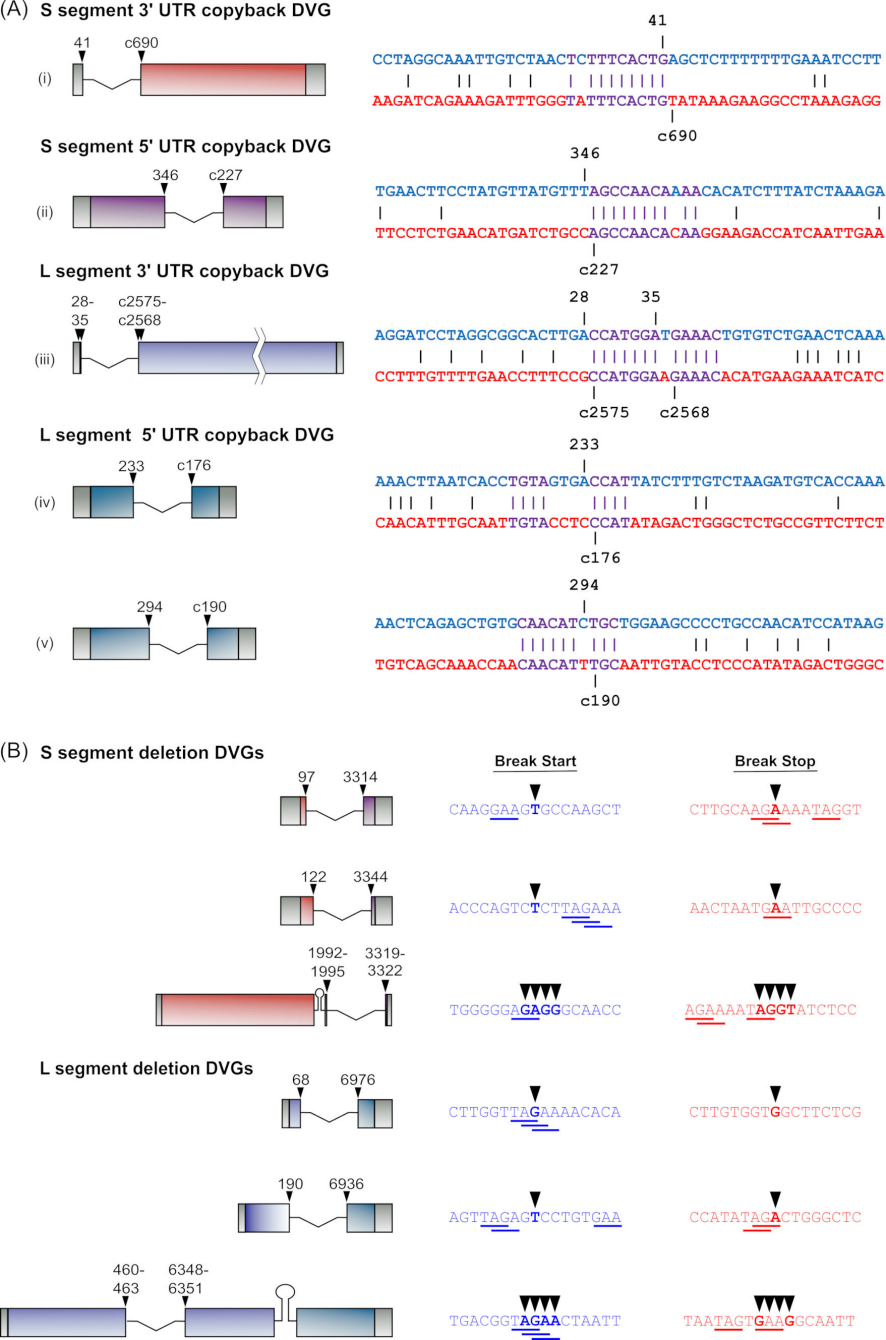
Sequence features associated with DVG formation. (**A**) Identity between template and complementary strand sequences surrounding break start/stops in highly abundant cb-DVGs. The schematics of highly abundant cb-DVGs are shown (left). The sequences flanking the break start and stop site (indicated by their nucleotide positions) are shown. The sequence containing the break start is shown in blue, while the complementary sequence showing the break stop is shown in red, with nucleotide identities indicated by dashes. Regions flanking the break start/stop sites that show a high degree of local identity are shown in purple. (**B**) Enrichment of specific nucleotide triplets in sequences surrounding the break start/stops of highly abundant del-DVGs. The schematics of highly abundant del-DVGs produced from the S segment or the L segment are shown (left). The sequences flanking the break start and stop sites (indicated by arrowheads) are shown with the sequence containing the break start in blue, and the sequence containing the break stop in red. Nucleotide triplet sequences that were enriched are shown inunderlined.

The observed hyper-prevalence of a few types of DVGs with specific pairs of break start/stops clearly suggests that a specific biological mechanism regulates their formation at these positions. We, therefore, more closely examined the sequences surrounding these sites to identify common features that might be of mechanistic significance. An analysis of the most common del-DVGs (Fig. 5) for sequence identity between break start/stop positions revealed no significant homology (Fig. S5). However, a similar analysis of the most prevalent cb-DVGs with a single set of break start/stops revealed clearly elevated levels of sequence identity surrounding the breakpoints (Fig. 11A). For the S segment 3′ UTR and 5′ UTR cb-DVGs, as well as the L-segment 3′ UTR cb-DVGs, these regions of homology were either immediately before or after the break points (11Ai through Aiii). In contrast, for the L-segment 5′ UTR cb-DVGs, the region of increased homology was more evenly centered around the break point, so that identical residues were found both before and after the break start/stop positions (Fig. 11Aiv and Av).

Given that regions of local sequence identity did not appear to be playing a role in the generation of del-DVGs (despite the fact that we also observed highly preferred sites for their formation), we further investigated a possible role of specific nucleotide triplet sequences in their formation, since this has been suggested to play a role in regulating DVG formation for other viruses. To this end, we analyzed all triplets present in a region spanning 8 nt before and after each break start/stop site for the most prevalent del-DVGs. Here we observed that three triplets, that is, TAG, AGA, and GAA, occurred in sequences flanking the break starts/stops in all six of these highly abundant del-DVGs (Fig. 11B; see Table S23 at https://zenodo.org/records/14900940). This represents a statistically improbable overrepresentation compared to both what would be expected assuming an equal distribution of triplet sequences within the genome and when taking into account the triplet frequency actually found within the TCRV genome (see Table S23 at https://zenodo.org/records/14900940). Intriguingly, these triplets also often occurred in overlapping combinations, that is, TAGA, AGAA, or TAGAA, and often directly at the break sites. In contrast, no significant enrichment of specific triplet sequences was observed for sequences flanking break start/stop sequences associated with cb-DVG generation (see Table S23 at https://zenodo.org/records/14900940).

## DISCUSSION

DVGs are defective byproducts of RNA virus replication that, unlike their parental standard viral genomes, contain modifications that make them incapable of supporting an infection on their own, and as such, they have traditionally been excluded from consideration with respect to virus biology. However, a growing body of evidence suggests that the production of DVGs can play a crucial role in determining infection outcome both by regulating virus infection, including during the establishment of persistence, as well as by helping to shape the host immune response to infection (reviewed in reference 12). In light of these contributions to virus biology, there is increasing appreciation regarding the need to better understand the generation and biological activities of such DVGs.

Currently, little is known about the structure or biological functions of DVGs during arenavirus infection, although their existence has long been suspected for LCMV, based on evidence for DI particle production, which appears to play a role in persistence both *in vitro* (23, 33) and *in vivo* (22). Support for the association of DVGs with both DI formation and persistence is provided by the accumulation of diversely sized shorter genome forms during *in vivo* infection (18), as well as the association of persistent virus infection of cell cultures with the generation of unusual DVG forms that either lack or contain additional nucleotides in the genome termini (19). With the exception of these DVGs with modified genome termini reported for LCMV, until recently, nothing was known regarding the actual detailed sequences of the DVGs produced by arenaviruses. However, recent work in this area has shown that not only does interference occur during acute infection of A549 cells at high MOIs, but it is also associated with the formation of a wide range of DVGs (24). Most notably, this analysis led to the identification of a highly prevalent S segment DVG with a small portion of the GPC gene and part of the IGR deleted, which the authors could show is specifically enriched in high passage stocks and has potent interfering activity, which it appears to exert by reducing the expression of GPC (24). In comparison to LCMV, less is known about the biological roles of DI particles and/or DVG formation during infection with other arenaviruses; however, limited evidence also suggests that these other arenaviruses can indeed also produce DI particles, and that their formation is associated with the accumulation of non-standard viral RNA products (20, 21, 24, 34). In particular, early work with TCRV showed that DI particles from persistently infected BHK cells lost their standard S and L segments while acquiring five to six discrete smaller RNA species, at least one of which was likely derived from the L segment based on its size (which was greater than that of the full-length S segment) (20).

To better understand the formation of DVGs during New World arenavirus infection, we employed a classical approach to enrich DVG content in viral stocks by serial passage at high MOI. As expected, this resulted in accumulation of subgenomic viral RNAs at the expense of full-length virus genomes, and this, in turn, correlated with reduced viral titers in these DVG-enriched stocks (Fig. 2). For one of the larger DVGs that was identified, with a size of approximately 2.1 kb, direct Sanger sequencing of the PCR product showed that it is missing the majority of the GPC ORF (Fig. 3). Interestingly, studies looking at persistently arenavirus-infected cell cultures have previously reported that such cells can continuously express NP (18, 20, 35–41), but show reduced or no mature glycoprotein on the cell surface (20, 35, 36, 40–42). Such results might be explained by the presence of a DVG in these persistently infected cells, similar to the S segment-derived 2.1 kb DVG we report in this study. With an intact NP ORF (and an L segment with an intact L ORF), these DVGs would be able to continuously replicate and express NP but would not support infectious particle production, unless GPC was supplied *in trans* (e.g., through reinfection with a standard virus). Notably, a previous study examining the sequences of DVGs produced during LCMV and Candid#1 infection also detected products of the S segment in which large parts of either the NP or GPC gene were deleted (24), some of which then closely resemble the S segment 2.1 kb DVG we identified for TCRV (Fig. 3), supporting that such DVGs are a common feature of infection with several different arenaviruses.

As reported by others, the subgenomic RNA populations produced during arenavirus infections are often highly heterogeneous (18, 21), and this is also consistent with what we observed here (Fig. 2; Fig. S2). To overcome challenges in sequencing these complex product pools, we performed nanopore-based sequencing to allow long single-read sequencing of entire DVG products following amplification by RT-PCR using primers specific for the genome ends. Based on our analysis, we could show that TCRV infection produces both del-DVGs and cb-DVGs from both the S and L genome segments. Interestingly, we found that the prevalence of these different DVG species differed markedly between the two segments, with the S segment producing a similar proportion of del-DVGs and cb-DVGs reads (61% vs 39%), while the L segment produced vastly more del-DVGs reads (i.e., 97% vs 3%) (Fig. S3). While this relatively small proportion of L segment cb-DVGs appears to be consistent with what was recently reported for LCMV, that study also observed only a very small proportion (<1%) of S segment cb-DVGs compared to what we observed (24). It is possible that the enhanced presence of S segment cb-DVGs in our samples represents an inherent difference between TCRV and LCMV (e.g., as New World and Old World arenaviruses, respectively) (24). However, since the same previous study also showed similarly low levels of cb-DVG formation using the New World arenaviruses Candid#1 (the JUNV vaccine strain) and Paraná virus (24), it appears more likely that experimental considerations are involved. One possible explanation is that the previous study examined DVG formation in samples collected after a primary infection for 48 h (24), while our study focused on the use of serial passaging to enhance the accumulation of DVGs. As such, it will be imperative that future studies explore both the diversity of DVG production and the temporal dynamics of their emergence during infections performed under various experimental conditions (i.e., single-cycle vs. multi-passage infection, acute vs persistent infection of cultures, infection of dead-end vs natural host cells).

Analysis of the frequency with which individual breakpoints were used to generate the observed DVGs allowed us to identify hotspots for the generation of both del-DVGs and cb-DVGs. We found that these were disproportionately located close to the genome ends, and that this was the case regardless of whether the DVGs were derived from the S or L segment, or whether they were del-DVGs or cb-DVGs (Fig. 5 to 9). Use of these preferred breakpoints then correspondingly directed the generation of relatively small DVG products that for the S segment ranged in size from ca. 200 to 700 bp, while for the L segment they ranged from 200 to 2,500 bp. Interestingly, we did not observe abundant DVG populations corresponding to those recently reported for LCMV with deletions of the GPC gene end and a portion of the IGR (24). We considered that this may have been due, at least in part, to the fact that our workflow defined DVGs for further analysis as those sequences having breakpoints >100 nt (to reduce the risk that DVGs were identified as a result of minor sequencing or alignment errors); however, lowering this threshold did not appreciably alter the results (Fig. S6 through S9). Rather, for del-DVGs, the only more frequently used breakpoints we observed in the central portion of the genome corresponded to the same 2.1 kB product that we had previously characterized by Sanger sequencing. However, this product was still a clear minority population compared to del-DVGs with breakpoints in the first/last few hundred nucleotides of the genome (Fig. 5A), which resulted in DVG lengths that were mostly around 200 nt in length.

Although our passaging data already clearly suggested that DVG accumulation was associated with low virus titers (Fig. 2), the identification of highly abundant del-DVGs by our study offered the opportunity to also investigate their biological functions directly. Therefore, we analyzed the impact of del-DVG coexpression in both a minigenome system, which models viral RNA synthesis, and a trVLP assay, which allows analysis not only of viral RNA synthesis (using a longer minigenome closer in size to the naturally occurring S segment) but also packaging of viral RNAs into particles and their delivery into target cells. These data clearly indicated that del-DVGs derived from both the S and L segments can compete with genome analogs (i.e., minigenomes) for the viral replication machinery, and thereby limit the expression of the encoded protein(s) (Fig. 10A and B), and that their ability to do so is length-dependent. Furthermore, we found that this del-DVG-mediated inhibition takes place not only in the cell in which the DVGs are initially produced, but also in new target cells into which they can be introduced as a result of their efficient incorporation into viral particles, which occurs at the expense of the minigenome (Fig. 10B and C). Importantly, differences in the results obtained using naïve target cells and those pre-transfected to overexpress the components of the replication machinery (i.e., NP and L) appear to support that inhibition by these del-DVGs is occurring through competition for a limiting supply of the viral RNA machinery, since overexpressing these components largely abolishes the impact of del-DVG coexpression. As such, these data strongly support that several of the highly prevalent del-DVGs observed in our study are contributing to the negative impact of DVG accumulation on virus stock titers that we observed during virus passage.

In contrast to the approach based on nanopore sequencing used here, most studies of DVG formation to date have been based on classical short-read next-generation sequencing approaches (17, 24, 43–47). Therefore, it is worth noting that our own data appear to correspond well to those of a recent analysis of JUNV (Candid#1) DVG formation (24), in that both reveal abundant formation of short DVGs with breakpoints near the genome termini as well as DVGs in which the majority of a single ORF within the S segment is deleted (i.e., similar to our 2.1 kb S segment DVG). This is despite the fact that DVGs in these two studies were generated using different infection approaches (i.e., single-cycle infection vs. passaging), amplified and sequenced using different methods (NGS vs RT-PCR and MinION), and analyzed using different workflows. However, while such NGS-based approaches also allow sequencing at the single-molecule level and can identify individual breakpoints, their short read lengths mean that they are limited in their ability to provide insight into more complex structural rearrangements, such as the multiple breakpoint cb-DVGs that we report here (Fig. 6 to 9). We found that many of these structures contained both the 3′ and 5′ UTR, as well as complements thereof, while lacking the majority of the sequence information between these elements. As such, they appear to have been generated by a combination of internal deletion and copyback events. Interestingly, while our data set also includes a small number of full-length genome reads (i.e., 199 S segment reads), we never observe structures containing duplications of the entire genome. This suggests that the process of internal deletion is necessary for the formation of structures with duplicated genome termini, although it remains unclear whether these deletion and copyback events are occurring within a single cycle of replication or as separate successive events. Given that these DVGs exhibit extensive potential for the formation of dsRNA structure (Fig. 7 to 9), which can in some cases include the entire DVG sequence, these may be particularly relevant as ligands for immune sensing during virus infection, and thus the ability to detect them appears to be a specific advantage of our approach. Indeed, a particularly important biological role of arenavirus DVGs with extensive dsRNA structure is suggested by the recent observation that arenaviruses possess an unusual dsRNA-specific exonuclease activity encoded by the viral NP that has been suggested to degrade viral RNAs that could otherwise serve as PAMPs for the activation of diverse RNA sensors (25–31). Supporting this, recent work has also shown that dsRNA (potentially in the form of DVGs, and especially cb-DVGs) indeed accumulates much more robustly in the cytoplasm of New World arenavirus-infected cells than for Old World arenaviruses, and that this appears to be dependent on the virus’ exonuclease function (32, 48). This is then potentially also consistent with the role of exacerbated pro-inflammatory responses (i.e., cytokine storm) in the severe forms of disease associated with New World arenaviruses, but not Old World arenaviruses (reviewed in reference 49). However, again here, a lack of knowledge about the characteristics of such viral dsRNA products has so far limited our ability to directly investigate their role in the virus’ biology. The increasing availability of data, including our own, precisely describing the sequence characteristics of individual arenavirus DVGs now opens up the possibility to analyze their biological properties in detail, including with respect to roles as both competitors for the replication machinery, as we have already begun to do here in this study, and as immune agonists.

While for many viruses, there remains little known about what triggers the detachment/reattachment events that drive DVG formation at specific sites, a growing body of evidence suggests that both virus and host factors can play a role, and in particular that specific sequence and/or structural elements in the viral genome can contribute to the formation of different types of DVG (reviewed in reference 13). For the IGR del-DVGs that have previously been reported for LCMV, it has been suggested that these are generated as a result of the highly structured nature of this RNA region (24). However, given that the arenavirus genome is encapsidated by NP, which should limit secondary structure formation, it appears unlikely that such a mechanism would be playing a role in other regions of the genome. Nonetheless, our data clearly show strong preferences for the use of specific breakpoints within regions outside the IGR (Fig. 5 to 8). Here, we clearly observed that the generation of cb-DVGs was associated with high levels of local sequence identity in proximity to the breakpoints (Fig. 11A). Interestingly, something similar has been observed for positive-sense RNA viruses, where partial sequence homology between break starts and stops has led to the hypothesis that for these viruses, DVG generation is driven by homologous recombination (50, 51). And while such a mechanism might be considered unlikely for negative-sense RNA viruses (due to the encapsidation of their genomes within the ribonucleoprotein complex), it is important to note that arenaviruses are known to have undergone recombination in nature (i.e., to generate the Clade A/B viruses) (52), and thus a homologous recombination-driven DVG generation cannot be so easily excluded. In contrast, for del-DVGs, we observed that highly favored break points were associated with the presence of specific nucleotide sequences, that is, TAG, AGA, and GAA, and that these frequently also occurred in longer overlapping combinations, that is, TAGA, AGAA, or TAGAA (Fig. 11B). This then appears similar to what has been reported for RSV (17) and also influenza virus (16), where the presence of specific sequence motifs also seems to be associated with DVG formation. However, the fact that certain break start/stop combinations are also highly favored (e.g., for the S segment, the combinations 97::3314 and 122::3344 are frequently observed, whereas 97::3344 was never observed, and 122::3314 only very rarely) suggests that additional sequence and/or structural elements must play a role in the pairing of break starts and stops, although their nature remains elusive at this time. Nonetheless, taken together, this collective evidence for the existence of common sequence elements/features among genetically diverse virus families supports the hypothesis that the generation of DVGs, including by arenaviruses, is not a random process but is rather influenced by these local sequence features.

In addition to its scientific findings, the presented work establishes a new workflow for the analysis of sequencing data for the detection of del-DVGs, as well as cb-DVGs generated from either genome end, based on long single-read sequencing data, such as is generated by nanopore sequencing (Fig. 4). Indeed, to the best of our knowledge, the only tool currently available to handle long-read nanopore sequencing data is BBMAP v36; however, it does not allow for the analysis of cb-DVGs (53). Almost all other currently existing analysis tools for DVG analysis are only designed to handle short-read Illumina (or 454) next-generation sequencing data (17, 44–47, 54). Notably, this severely limits the ability of these approaches to detect multiple-breakpoint DVGs, which our study identified as composing a substantial proportion of cb-DVG reads from both genome segments. Furthermore, only a few of these existing DVG analysis tools are capable of detecting both del-DVGs and cb-DVGs (i.e., VODKA2 [47], DI-tector [46] and DVG-profiler [44]), and even then they contain assumptions that may bias them, for instance, in favor of the detection of only 5′ UTR copyback reads (i.e., those starting from the 3′ genome end of the cRNA, proximal to the arenavirus GPC gene) (47). While this is based on the observation that 5′ trailer (UTR) cb-DVGs appear to be more prevalent for negative-sense RNA viruses, it is not clear that this assumption is valid for ambisense viruses like the arenaviruses. In contrast, our approach based on alignment to reference genomes with both duplicated complementary 3′ UTRs or 5′ UTRs allows equally for the detection of cb-DVGs formed starting from either genome terminus. However, in this study, we initially imposed the additional restriction that only gaps between break start and stop sites of >100 bp were reported, to conservatively exclude the possibility of false positives due to minor mismatches during alignment (including due to sequence errors, which are still potentially a greater challenge for nanopore sequence data than for short-read next-generation sequencing methods). While this potentially limits our ability to detect del-DVGS with extremely short deletions like those found in the IGR of LCMV (24), repeating the analysis with thresholds of 50 or 25 nt did not result in notable changes to our data (Fig. S6 through S9). Given its ability to handle nanopore sequencing data and to detect not only del-DVGs but also both 3′ and 5′ UTR cb-DVGs, as well as its fundamentally different assumptions and biases compared to existing tools, we suggest that this workflow represents a valuable complement to the currently available resources for the study of DVGs.

Furthermore, it is worth noting that a potential caveat of current DVG sequencing approaches is their reliance on RNA to DNA conversion and DNA amplification prior to or during sequencing. In particular, deletions involving the highly structured arenavirus IGR have been reported to occur as a product of amplification by RT-PCR (55). While this potential to generate aberrant products through RT-PCR amplification itself remains a significant challenge to many studies that look at DVG formation during a single round of infection, we avoid this issue by focusing on DVGs that only accumulate over sequential rounds of passaging. Importantly, in these experiments, we do not see significant accumulation of subgenomic products during RT-PCR amplification with non-passaged virus stocks (which contain higher levels of full-length genome template) (Fig. 2), but rather these only occur as a product of passaging (and at the expense of the full-length S segment), thus strongly suggesting that the formation of these structures has a biological basis and is not an RT-PCR artifact. Furthermore, upon passaging of our p20 stock, we observed spontaneous reversion from a high DVG content/low titer (p22) to a low DVG content/high titer (p23) state (Fig. 2). Such an event is consistent with an interfering nature of these DVGs and with competition between DI and standard viruses, but inconsistent with them being RT-PCR artifacts. Nonetheless, these amplification steps remain a technical challenge for many of these kinds of studies, and especially those focused on studying DVG generation during a single cycle of infection. While direct RNA sequencing using nanopore technology has recently been used as a supplemental approach to avoid this issue and validate individual findings from classical next-generation sequencing-based studies of DVG formation (24), it is still currently not a viable option for *de novo* sequence determination, due to problematically high levels of sequencing errors that arise as a result of extensive RNA modifications (56). Nonetheless, it is to be hoped that further technical improvements may make direct nanopore sequencing of RNA a viable approach for such studies in the future. At that time, the scripts that we have developed as part of our analysis pipeline could be seamlessly adapted to such data by substituting an RNA rather than a DNA reference sequence.

Taken together, our work presents not only a novel workflow for the analysis of DVGs present in nanopore sequencing data but also provides insight regarding DVG diversity during TCRV infection by identifying the most highly enriched DVG products of both genome segments. Based on this information, we could demonstrate a length-dependent competition for interaction with the viral RNA synthesis machinery by several short del-DVGs identified in our study. Finally, the identification of a substantial number of highly enriched del-DVG and cb-DVG species has allowed us to uncover genome sequence features that are associated with their production. As such, our work will not only enable future research investigating the biological activities of specific arenavirus DVGs, but also open up the possibility to regulate their production (i.e., by modifying the sequence elements associated with their formation) to study their biological impact and modulate infection outcome.

## MATERIALS AND METHODS

### Cells and viruses

TCRV (strain TRVL-11573) was kindly provided by Dan Kolakofsky and Dominique Garcin (University of Geneva). Vero76 cells (African green monkey kidney; Collection of Cell Lines in Veterinary Medicine [CCLV]-RIE 0228) were maintained in Dulbecco’s Modified Eagle Medium (DMEM) + 10% fetal calf serum (FCS) and 100 U/mL penicillin, and 100 µg/mL streptomycin (P/S). Cells were cultured at 37°C in 5% CO_2_.

### Virus passaging

Vero76 cells were seeded for 80%–90% confluence in 12-well plates. Initial infections were performed in triplicate at MOI = 0.5 using TCRV stock virus diluted in DMEM without FCS. After 1 h at 37°C, the inoculum was exchanged for 2 mL DMEM + 2% FCS. After incubation for 7 days, 500 µL of undiluted cell culture supernatant was used to infect freshly seeded Vero76 cells using the same approach as for the initial infections. The remaining supernatant was stored at −20°C until all samples had been collected before being processed as described in the sections below.

### Plaque assay

Vero 76 cells were seeded into 12-well plates for a confluence of ~90% on the following day. They were infected for 1 h at 37°C with serial dilutions of virus stocks collected at the indicated passages. After 1 h, the inoculum was removed, and an overlay composed of a 1:1 mixture of 2× MEM + 4% FCS, and 1.4% agarose was added. Plates were fixed and stained 10 days post-infection by incubation with 10% formalin containing crystal violet.

### RT-PCR amplification for Sanger sequencing

RNA was isolated from serially passaged TCRV p1, p5, p10, p15, and p20 samples (three independent replicates each) using the QIAamp Viral RNA Mini Kit (Qiagen). cDNA was generated using SuperScript III Reverse Transcriptase (Invitrogen) with a universal L and S segment 3′ UTR primer (CGCACAGTGGATCCTAGGC). For initial analysis of DVG accumulation, cDNA was then amplified by PCR using S segment-specific genome end primers (S segment 3′ UTR Fwd: GGCAAATTGTCTAACTCTTTCACTGAG, S segment 5′ UTR Rev: CCTAGGCATTTCTTGACCATATTTGC). The resulting PCR products were then separated by agarose gel electrophoresis and stained with ethidium bromide. Subgenomic products of >1 kb were gel extracted using the NucleoSpin Gel and PCR Clean-up Kit (Macherey-Nagel) before being sent for commercial Sanger sequencing (Eurofins). The resulting data were analyzed by alignment to a TCRV S segment reference sequence (GenBank Accession number: MT081316 [57]) using Geneious Prime (Biomatters).

### RT-PCR amplification for nanopore sequencing

For nanopore sequencing of DVGs, separate PCRs using cDNA (generated as described above from p20 virus samples; three replicates) were performed for each genome segment using the iProof High-Fidelity PCR Kit (Bio-Rad) with segment-specific genome end primers (S segment primers, as above; L segment 3′ UTR Fwd: ATCCTAGGCGGCACTTGACC, L segment 5′ UTR Rev: CCTAGGCGTTACGTGCACTC). To amplify del-DVGs, both forward and reverse primers were used for amplification of the respective segment, while for the detection of cb-DVGs (which contain only one genome end and its complement), PCR was run with either only the forward or only the reverse primer. All PCR products were purified using the NucleoSpin Gel and PCR Clean-up Kit (Macherey-Nagel) prior to further analysis.

### Nanopore library preparation and sequencing

Following PCR amplification, a nanopore sequencing run (Oxford Nanopore Technologies) was prepared using the 1D2 kit according to the manufacturer’s protocol (version LSD_9032_v11_revO_23Mar2017). Briefly, an equal mass of PCR product from each reaction was combined for a total of 1 µg, and nuclease-free water was added to a final volume of 45 µL. No barcoding was implemented in this approach, but a linker sequence, as well as an adapter (to allow binding of the DNA to the nanopores), was added to the DNA in successive reactions, with purification of the DNA using magnetic AMPure XP beads between the reactions. Finally, the prepared sample was loaded onto a MinION sequencer containing a FLO-MIN107 R9 flow cell, also as per the manufacturer’s instructions. The flow cell used was confirmed to have 1,360 functional pores. For sequencing, the MinKNOW software (version 2.2.15) with integrated live base-calling was used. The sequencing run was allowed to proceed for 16 h.

### Analysis of nanopore sequencing data

Data were analyzed according to the workflow shown in Fig. 4 using the scripts detailed in the supplemental information (Supplementary Methods) running under Ubuntu 18.04.6. Following this approach, the obtained sequences were first divided into S-segment or L-segment sequences by searching for the primer sequences used in PCR amplification at positions close to the genome termini using flexbar (v3.1) (58). Flexbar parameters were optimized for this purpose using a test set of 400 sequences and gave a sensitivity of 89% and a specificity of 99% when compared manually to alignments of the reads to reference sequences for the S or L segment (GenBank accession numbers: S segment, MT081316; L segment, MT081317 [57]) using Geneious Prime (Biomatters).

Reads that mapped to a specific genome segment (i.e., S or L) were further sorted based on local alignment to four different reference sequence templates using lastal (version 921) (59) with parameters that had been optimized for nanopore data (60). These four templates corresponded to (i) the vRNA-sense genome segment, (ii) the cRNA-sense genome segment, (iii) a vRNA-cRNA concatemer, and (iv) a cRNA-vRNA concatemer. For each read, the number of local matches to each of the four templates (i.e., the vRNA, cRNA, vRNA_cRNA, or cRNA_vRNA match counts) was determined, and this information was used to deduce the DVG type. If there was only a single match, the read was discarded from the analysis, since it then corresponded to a full-length genome rather than a DVG. In all other cases, if the vRNA or cRNA match count was as high or higher than all other counts, then the read was considered a del-DVG. Importantly, however, while vRNA and cRNA reads were aligned and analyzed separately in the bioinformatics workflow, due to the PCR amplification step, a distinction between vRNA and cRNA input material is not possible, as either RNA species can be amplified into a double-stranded PCR product, of which one random strand was then sequenced through the nanopore. In contrast, if the vRNA_cRNA or cRNA_vRNA match count was higher than the vRNA and cRNA match count, then the read was identified as a cb-DVG. Here, the order of the local alignments can only correspond to either a vRNA_cRNA or a cRNA_vRNA reference sequence, and thus the order of these local alignments was used to determine whether reads corresponded to 3′ or 5′ UTR cb-DVGs.

### Analysis of break points

Once sorted into categories, reads and reference sequences for each local alignment were trimmed to remove barcodes and linker sequences from the reads and to ensure consistent ends. A global alignment was then performed using the appropriate reference template (i.e., vRNA, cRNA, cRNA_vRNA, or cRNA_cRNA) and the Needle-Wunsch algorithm as implemented in needle (version: EMBOSS:6.6.0.0) (61) with a high gap open penalty (i.e., 50) but a very low gap extend penalty (i.e., 0.000000001) to allow the algorithm to tolerate the large deletions that are expected for many DVGs. The output of the Needle-Wunsch algorithm was then examined for breakpoints, with a gap of at least 100 nt being defined as a break. This conservative cutoff was chosen to avoid false-positive detection of breaks due to sequencing inaccuracies; however, subsequent analysis showed that the results remained virtually unchanged if cutoffs of 50 nt or 25 nt were used (Fig. S6 through S9). A “break start” was defined as the last nucleotide of a reference that aligns to a read before the deletion, while a “break stop” was the first nucleotide of the read that again aligns to the reference sequence after the deletion. Break start and stop points for each DVG were then extracted and further analyzed in Excel (Microsoft). Since the DVG products analyzed were generated by PCR (and thus both strands are present for any given DVG), the break positions for del-DVGs in cRNA orientation were converted into their vRNA equivalents, and the data were combined.

### Minigenome assay

Huh7 cells were seeded into 12-well plates for a confluency of ~60% on the next day. They were then transfected with a nanoluciferase (nLuc)-expressing monocistronic TCRV minigenome (125 ng [62]), pCAGGS-T7 (125 ng), pCAGGS-TCRV L (100 ng), pCAGGS-TCRV NP (25 ng), and pCAGGS-firefly-luciferase (FF, 50 ng; as a transfection control) using Transit-LT1 with a ratio of 3 µL to 1 µg of DNA. As controls, samples without pCAGGS-L (-L) and with only half the amount of transfected pCAGGS-NP and pCAGGS-L (+L 0.5×) (i.e., 50 ng pCAGGS-L and 12.5 ng pCAGGS-NP) were prepared. To assess the inhibitory activity of DVGs, 125 ng of the indicated DVG was additionally transfected. Total plasmid amounts were equalized between samples by adding empty pCAGGS. After 24 h, the medium on the transfected cells was exchanged against fresh DMEM + 10% FCS, and after a further 24 h, the cells were lysed in 1% Triton-X100, and luciferase activity was measured using Nano-Glo (for nLuc) and Beetle Juice (for FF) reagents in a Glomax Multi microplate reader. Normalized luciferase values were calculated based on the nLuc (viral RNA synthesis) and FF (host cell RNA synthesis) values.

### Transcription and replication-competent virus-like particle assay

Huh7 cells were seeded as described above for the minigenome assay. These cells (i.e., p0 cells) were transfected with a bicistronic TCRV minigenome expressing the GPC linked by a T2A sequence to nLuc (GPC-T2A-nLuc) and Z (250 ng [62]), pCAGGS-T7 (125 ng), pCAGGS-TCRV L (100 µg), pCAGGS-TCRV NP (25 ng), and pCAGGS-FF (50 ng) using Transit-LT1 at a ratio of 3 µL to 1 µg of DNA. After 24 h, the medium on the transfected cells was exchanged against fresh DMEM + 10% FCS, and after a further 48 h, supernatants were harvested, and the p0 cells were lysed in 1% Triton-X100, and luciferase activity was measured using Nano-Glo (for nLuc) and Beetle Juice (for FF) reagents in a Glomax Multi microplate reader. The supernatants were cleared of debris by centrifugation at 500 × *g* for 5 min and used to infect fresh Huh7 cells at a confluence of ~50% in 96-well plates (i.e., p1 cells) in triplicate. These p1 cells were either untransfected (i.e., naïve) or had been transfected the day before (i.e., pre-transfected) with pCAGGS-TCRV NP (15 ng) and pCAGGS-TCRV L (55 ng) per well using Transit-LT1. After 72 h, the p1 cells were lysed in 1% Triton-X100, and luciferase activity was measured using Nano-Glo (for nLuc) in a Glomax Multi microplate reader. Normalized luciferase values were calculated for p0 cell lysates based on the nLuc (viral RNA synthesis) and FF (host cell RNA synthesis) values, while only nLuc (viral RNA synthesis) values are shown for p1 cells.

## Data Availability

The original sequencing reads are deposited in the European Nucleotide Archive (https://www.ebi.ac.uk/ena/browser/home) under the study accession number PRJEB85984. The supplemental tables and global alignments are deposited in Zenodo (zenodo.org) under the DOI https://doi.org/10.5281/zenodo.14900940. All other data are part of this article and its supplemental material.

## SUPPLEMENTAL MATERIAL

Supplemental figures jvi.01978-25-s0001.pdf Figures S1 to S9.

Supplemental text jvi.01978-25-s0002.pdf Scripts used for data analysis.

ASM does not own the copyrights to Supplemental Material that may be linked to, or accessed through, an article. The authors have granted ASM a non-exclusive, world-wide license to publish the Supplemental Material files. Please contact the corresponding author directly for reuse.
