# Supplementary material for: Molecular characterization of arenavirus defective viral genomes reveals sequence features associated with their formation
Source: J Virol. 2025 Dec 9;100(1):e01978-25. doi: 10.1128/jvi.01978-25 (PMC12817923; doi:10.1128/jvi.01978-25)
Supplement: Supplemental figures — Figures S1 to S9. [file jvi.01978-25-s0001.pdf]

|  |  | Replicate 1 |  |  |  |  |  |  |  | Replicate 2 |  |  |  |  |  |  |  | Replicate 3 |  |  |  |  |  |  |  |
|---|---|---|---|---|---|---|---|---|---|---|---|---|---|---|---|---|---|---|---|---|---|---|---|---|---|
|  |  | p5 |  | p10 |  | p15 |  | p20 |  | p5 |  | p10 |  | p15 |  | p20 |  | p5 |  | p10 |  | p15 |  | p20 |  |
|  |  | Nucleotide | Amino Acid | Nucleotide | Amino Acid | Nucleotide | Amino Acid | Nucleotide | Amino Acid | Nucleotide | Amino Acid | Nucleotide | Amino Acid | Nucleotide | Amino Acid | Nucleotide | Amino Acid | Nucleotide | Amino Acid | Nucleotide | Amino Acid | Nucleotide | Amino Acid | Nucleotide | Amino Acid |
| 3'-UTR | 1-77 |  |  |  |  |  |  |  |  |  |  |  |  |  |  |  |  | (C46T) | n.a. |  |  |  |  | (C46T) | n.a. |
|  |  | (Δ47T) | n.a. | Δ47T | n.a. | Δ47T | n.a. | (Δ47T) | n.a. | (Δ47T) | n.a. | Δ47T | n.a. | Δ47T | n.a. | (Δ47T) | n.a. |  |  | Δ47T | n.a. | Δ47T | n.a. |  |  |
| NP | 78-1790 |  |  | A486G | S>G | A486G | S>G | (A486G) | S>G |  |  |  |  |  |  |  |  |  |  |  |  |  |  |  |  |
|  |  |  |  |  |  |  |  |  |  |  |  | G521A | Q>Q | G521A | Q>Q | (G521A) | Q>Q |  |  |  |  |  |  |  |  |
|  |  |  |  |  |  |  |  |  |  |  |  |  |  |  |  |  |  |  |  |  |  |  |  | (G681T) | G>C |
|  |  | (G1110A) | A>T |  |  |  |  |  |  |  |  |  |  |  |  |  |  |  |  |  |  |  |  |  |  |
|  |  |  |  |  |  |  |  |  |  |  |  |  |  |  |  |  |  |  |  | A1134G | K>E | (A1134G) | K>E |  |  |
|  |  | (A1620G) | N>D | A1620G | N>D | A1620G | N>D | (A1620G) | N>D |  |  |  |  |  |  |  |  |  |  |  |  |  |  |  |  |
|  |  |  |  |  |  |  |  | (A1723G) | K>R |  |  |  |  |  |  |  |  |  |  |  |  |  |  |  |  |
|  |  |  |  |  |  |  |  |  |  |  |  | A1749G | K>E | A1749G | K>E | (A1749G) | K>E |  |  |  |  |  |  |  |  |
| IGR | 1791-1902 |  |  |  |  |  |  |  |  |  |  | (A1870G) | n.a. |  |  |  |  |  |  | (A1870G) | n.a. |  |  |  |  |
| GPc | 1903-3354 |  |  |  |  |  |  | G2065A | F>F |  |  |  |  |  |  |  |  |  |  |  |  |  |  |  |  |
|  |  |  |  |  |  |  |  |  |  |  |  | T2103C | I>V | T2103C | I>V |  |  |  |  |  |  |  |  |  |  |
|  |  |  |  |  |  |  |  |  |  |  |  | G2263A | H>H | G2263A | H>H |  |  |  |  |  |  |  |  |  |  |
|  |  |  |  |  |  |  |  |  |  |  |  | (G2705A) | P>L | (G2705A) | P>L |  |  |  |  |  |  |  |  |  |  |
|  |  |  |  |  |  |  |  |  |  |  |  |  |  |  |  |  |  |  |  | (G3073A) | A>A | G3073A | A>A |  |  |
|  |  | (C3249A) | V>L | C3249A | V>L | C3249A | V>L | (C3249A) | V>L |  |  |  |  |  |  |  |  |  |  | C3249T | V>M | C3249T | V>M | (C3249T) | V>M |
| 5'-UTR | 3355-3422 |  |  |  |  |  |  |  |  |  |  |  |  |  |  |  |  |  |  |  |  |  |  |  |  |

|  |  | Replicate 1 |  |  |  |  |  |  |  | Replicate 2 |  |  |  |  |  |  |  | Replicate 3 |  |  |  |  |  |  |  |
|---|---|---|---|---|---|---|---|---|---|---|---|---|---|---|---|---|---|---|---|---|---|---|---|---|---|
|  |  | p5 |  | p10 |  | p15 |  | p20 |  | p5 |  | p10 |  | p15 |  | p20 |  | p5 |  | p10 |  | p15 |  | p20 |  |
|  |  | Nucleotide | Amino Acid | Nucleotide | Amino Acid | Nucleotide | Amino Acid | Nucleotide | Amino Acid | Nucleotide | Amino Acid | Nucleotide | Amino Acid | Nucleotide | Amino Acid | Nucleotide | Amino Acid | Nucleotide | Amino Acid | Nucleotide | Amino Acid | Nucleotide | Amino Acid | Nucleotide | Amino Acid |
| 3'-UTR | 1-30 | (C29T) | n.a. |  |  |  |  |  |  |  |  |  |  | (C29T) | n.a. | C29T | n.a. |  |  |  |  |  |  | (C29T) | n.a. |
| L | 31-6663 |  |  |  |  |  |  |  |  |  |  |  |  |  |  | (C937A) | L>I |  |  |  |  |  |  |  |  |
|  |  | (T1035C) | N>N | T1035C | N>N | T1035C | N>N | T1035C | N>N |  |  |  |  |  |  |  |  |  |  |  |  |  |  |  |  |
|  |  |  |  |  |  |  |  |  |  |  |  | (G1080A) | L>L |  |  |  |  | (G1080A) | L>L | (G1080A) | L>L | (G1080A) | L>L | (G1080A) | L>L |
|  |  |  |  |  |  |  |  |  |  |  |  | (C1203T) | D>D |  |  |  |  |  |  |  |  |  |  |  |  |
|  |  |  |  |  |  |  |  |  |  |  |  | (T1300A) | S>T |  |  |  |  |  |  |  |  |  |  |  |  |
|  |  |  |  |  |  |  |  |  |  |  |  |  |  | (A1878G) | L>L |  |  |  |  |  |  |  |  |  |  |
|  |  |  |  |  |  |  |  |  |  |  |  |  |  |  |  |  |  |  |  |  |  | T2301C | D>D | (T2301C) | D>D |
|  |  |  |  | (C2337T) | D>D | C2337T | D>D | C2337T | D>D |  |  |  |  |  |  |  |  |  |  |  |  |  |  |  |  |
|  |  |  |  | (G2353A) | G->G |  |  |  |  |  |  |  |  |  |  |  |  |  |  |  |  |  |  |  |  |
|  |  |  |  |  |  |  |  |  |  |  |  |  |  |  |  |  |  |  |  |  |  |  |  | (T2361A) | N>K |
|  |  |  |  | (A2380G) | I->V |  |  |  |  |  |  |  |  |  |  |  |  |  |  |  |  |  |  |  |  |
|  |  |  |  | (A2436G) | P>P |  |  |  |  |  |  |  |  |  |  |  |  |  |  |  |  |  |  |  |  |
|  |  |  |  | (A2446G) | N>D |  |  |  |  |  |  |  |  |  |  |  |  |  |  |  |  |  |  |  |  |
|  |  |  |  | (T2481G) | N>K |  |  |  |  |  |  |  |  |  |  |  |  |  |  |  |  |  |  |  |  |
|  |  |  |  | (G2491A) | V->I |  |  |  |  |  |  |  |  |  |  |  |  |  |  |  |  |  |  |  |  |
|  |  |  |  | (T2569A) | S->T |  |  |  |  |  |  |  |  |  |  |  |  |  |  |  |  |  |  |  |  |
|  |  | (A2603G) | Y->C | A2603G | Y->C | A2603G | Y->C | A2603G | Y->C |  |  |  |  |  |  |  |  |  |  |  |  |  |  |  |  |
|  |  |  |  |  |  |  |  |  |  |  |  |  |  |  |  | (C2735T) | A>V |  |  | (C2735T) | A>V | (C2735T) | A>V |  |  |
|  |  | (T2748C) | N>N | T2748C | N>N | T2748C | N>N | T2748C | N>N |  |  |  |  |  |  |  |  |  |  |  |  |  |  |  |  |
|  |  |  |  |  |  |  |  |  |  |  |  |  |  |  |  |  |  |  |  | (A2768T) | Q>L | A2768T | Q>L | (A2768T) | Q>L |
|  |  |  |  |  |  |  |  |  |  |  |  | (A2776T) | M>L |  |  |  |  |  |  |  |  |  |  |  |  |
|  |  |  |  |  |  |  |  |  |  |  |  |  |  |  |  |  |  |  |  |  |  | (A3453G) | I>M | (A3453G) | I>M |
|  |  |  |  |  |  |  |  |  |  |  |  |  |  |  |  |  |  |  |  |  |  |  |  | (G4365A) | L>L |
|  |  |  |  |  |  |  |  |  |  |  |  |  |  | (G4631A) | S>N |  |  |  |  |  |  |  |  |  |  |
|  |  |  |  |  |  |  |  |  |  |  |  |  |  |  |  |  |  |  |  | (T5223C) | G>G | (T5223C) | G>G |  |  |
|  |  |  |  |  |  |  |  |  |  |  |  |  |  | A5413G | R>G | (A5413G) | R>G |  |  |  |  |  |  |  |  |
|  |  |  |  | (G5452A) | A>T | G5452A | A>T | G5452A | A>T |  |  |  |  |  |  |  |  |  |  |  |  |  |  |  |  |
|  |  |  |  |  |  |  |  |  |  |  |  |  |  |  |  |  |  |  |  | (A5568G) | L>L | A5568G | L>L | (A5568G) | L>L |
|  |  |  |  |  |  |  |  |  |  |  |  | (G5595A) | S>S |  |  |  |  |  |  |  |  |  |  |  |  |
|  |  |  |  |  |  |  |  |  |  |  |  | (C5746G) | L>V |  |  |  |  |  |  |  |  |  |  |  |  |
|  |  |  |  |  |  |  |  |  |  |  |  | (A5753G) | D>G |  |  |  |  |  |  |  |  |  |  |  |  |
|  |  |  |  |  |  |  |  |  |  |  |  | (T5756G) | V>G |  |  |  |  |  |  |  |  |  |  |  |  |
|  |  |  |  | T5976C | D>D | T5976C | D>D | T5976C | D>D |  |  |  |  |  |  |  |  |  |  |  |  |  |  |  |  |
|  |  |  |  |  |  |  |  |  |  |  |  | (G6010A) | D>N | (G6010A) | D>N |  |  | (G6010A) | D>N | (G6010A) | D>N | G6010A | D>N | (G6010A) | D>N |
|  |  |  |  |  |  |  |  |  |  |  |  |  |  |  |  |  |  |  |  |  |  |  |  | (A6130G) | M>V |
|  |  |  |  |  |  |  |  |  |  |  |  |  |  |  |  |  |  |  |  | (T6203C) | L>S | T6203C | L>S | (T6203C) | L>S |
|  |  |  |  | (C6189T) | S>S | C6189T | S>S | C6189T | S>S |  |  |  |  |  |  |  |  |  |  |  |  |  |  |  |  |
|  |  |  |  |  |  |  |  |  |  |  |  |  |  |  |  |  |  |  |  |  |  |  |  | (G6330A) | L>L |
| IGR | 6664-6746 |  |  |  |  | (A6692C) | n.a. |  |  |  |  |  |  |  |  |  |  |  |  |  |  |  |  |  |  |
| Z | 6747-7034 |  |  |  |  |  |  |  |  |  |  |  |  |  |  |  |  |  |  | (C6937G) | S>T |  |  |  |  |
|  |  |  |  |  |  |  |  |  |  | (G6939C) | P>P |  |  |  |  |  |  |  |  |  |  |  |  |  |  |
|  |  |  |  |  |  |  |  |  |  | (T6969C) | Q>Q |  |  |  |  |  |  |  |  |  |  |  |  |  |  |
| 5'-UTR | 7035-7103 |  |  |  |  |  |  |  |  |  |  |  |  |  |  |  |  |  |  |  |  |  |  |  |  |

**Supplementary Figure S1. Accumulation of mutations in the TCRV genome during serial passage.** RNA was isolated from three independently generated replicates of TCRV serially passaged in Vero 76 cells after 5 (p5), 10 (p10), 15 (p15), or 20 (p20) passages, as indicated. Overlapping genome fragments covering the complete viral genome were amplified by RT-PCR and subjected to Sanger sequencing. The obtained sequence was then compared to the sequence of the starting virus stock. Mutations resulting in silent changes within the amino acid sequence are indicated in grey, while missense mutations in the coding sequence and changes in non-coding sequences are indicated in orange. Changes shown in light orange are only found in a single replicate, while those shown in dark orange are found in multiple replicates. Further, changes indicated in brackets were present only as minority sequence populations, while those without brackets were present in the majority sequence.

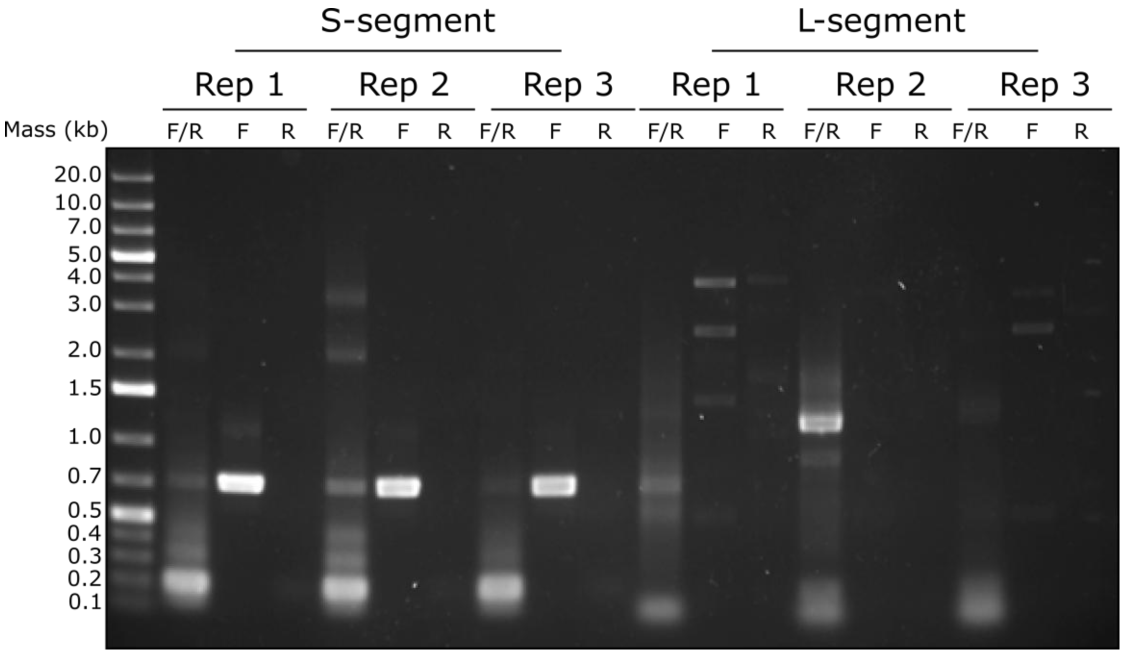


**Supplementary Figure S2. Analysis of TCRV DVG species by RT-PCR.** The RNA of three independently generated replicates of TCRV p20 (i.e. TCRV serial passaged 20 times in Vero 76 cells) was isolated from cell supernatant, reverse transcribed with a universal genome end primer and amplified with L and/or S segment specific genome end primers, as indicated. PCRs were performed with forward (3' UTR specific) and reverse (5' UTR specific) primers (F/R), the forward primer only (F), or the reverse primer only (R), as indicated. The full-length S segment has an expected size of 3.4 kb, while the full-length L-segment has an expected size of 7.1 kb.

(A)

| Category | | Reads |
|---|---|---|
| Arenavirus-Specific | | 581,781 |
| S segment | Total | 395,736 |
| | DVGs | 306,537 |
| | Deletion DVGs | 187,040 |
| | Copyback DVGs | 119,497 |
| | 3’ UTR Copyback | 117,811 |
| | 5’ UTR Copyback | 1,686 |
| L segment | Total | 186,045 |
| | DVGs | 162,297 |
| | Deletion DVGs | 157,786 |
| | Copyback DVGs | 4,511 |
| | 3’ UTR Copyback | 2,428 |
| | 5’ UTR Copyback | 2,083 |

(B)

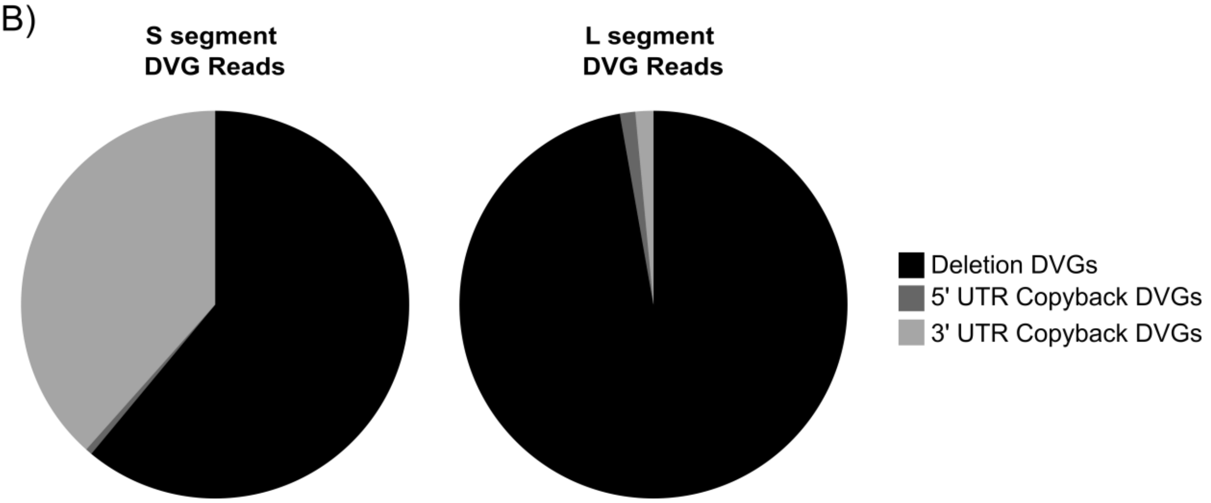


**Supplementary Figure S3. Categorization of sequencing reads.** (A) Number of DVG reads corresponding to different genome segments and DVG types. DVG reads were sorted into groups corresponding to the S segment or L segment and subjected to local alignment to determine whether they were internal deletion DVGs (black), 5’ UTR copyback DVGs (dark grey) or 3’ UTR copyback DVGs (light grey). (B) Graphical representation of read distribution. The number of reads sorted into each different DVG category is indicated as a percentage of reads for that segment.

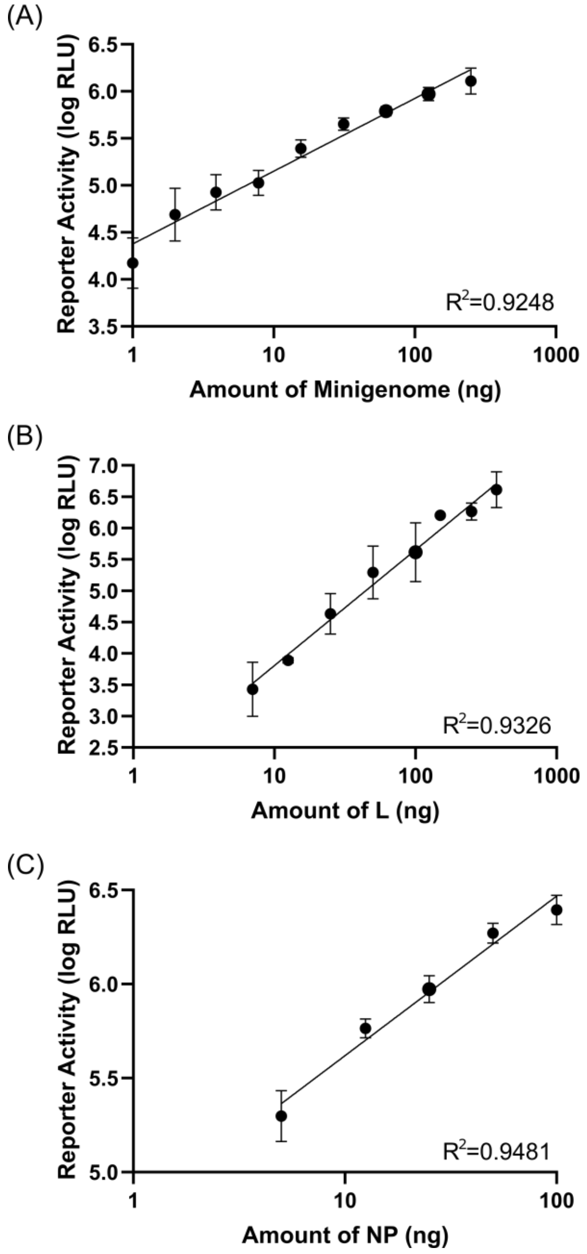


**Supplementary Figure S4. Optimization of minigenome assay conditions.** (A) Optimization of minigenome levels. Huh7 cells were transfected with the indicated amount of a monocistronic nanoluciferase-encoding TCRV minigenome, along with pCAGGS constructs encoding the T7 polymerase (125 ng), JUNV nucleoprotein (NP, 250 ng) and polymerase (L, 500 ng). pCAGGS-Firefly (FF; 50 ng) served as a transfection control. Cells were harvested 48 h later and measured for both nLuc (viral RNA synthesis) and FF (host cell RNA synthesis) activity. (B) Optimization of polymerase levels. Cells were transfected and reporter activity measured as described in (A) with the exception that the amount of pCAGGS-L was varied, as indicated, and the amount of minigenome transfected was held constant at 125 ng. (C) Optimization of nucleoprotein input. Cells were transfected and reporter activity measured as described in (A) with the exception that the amount of pCAGGS-NP was varied, as indicated, and the amount of minigenome transfected was held constant at 125 ng. The means and standard deviations of normalized reporter levels (nLuc/FF) shown represent the data from two independent experiments. The best-fit curve and coefficient of determination ($R^2$) are also shown.

**S segment 97-3314**

```
                                                97
                                                 |
TTTTTTGAAATCCTTGCTTTGATCGCCATAATGGCTCAATCCAAGGAAGTGCCAAGCTTCAGATGGACCCAGTCTCTTAGAAAGGGATTGAGTCAATTCA
|       |  |                 |               | |     |    |                    |||  |
TACAGATGAGGCTGACTGCCACCAATGCAATGTTGAGTGCTTCTTGCAAGAAAATAGGTATCTCCTGCATGAAACTAATGAATTGCCCCATGGTTGGGAG
                                                  |
                                                 3314
```

**S segment 122-3344**

```
                                                122
                                                 |
CCATAATGGCTCAATCCAAGGAAGTGCCAAGCTTCAGATGGACCCAGTCTCTTAGAAAGGGATTGAGTCAATTCACCCAAACAGTCAAGTCAGATATTTT
     |  |||   | |||| || |    | || | |   ||  |       | |        |  |        | |               | |
TGTTGAGTGCTTCTTGCAAGAAAATAGGTATCTCCTGCATGAAACTAATGAATTGCCCCATGGTTGGGAGTGATTCACCTGGTTCAGTTAGGCAAATATG
                                                  |
                                                3344
```

**L segment 68-6976**

```
                                                68
                                                 |
CGGCACTTGACCATGGATGAAACTGTGTCTGAACTCAAAGACTTGGTTAGAAAACACATTCCAAATAGGCATGAGTTTGCCCACCAGAAAGATGCCTTCC
|     |  ||   |        |   ||| || ||      |||    |    | |        |||         ||     |  |
TCCCATATAGACTGGGCTCTGCCGTTCTTCTAAATTCTGCAGCTTGTGGTGGCTTCTCGAGGTTGTTAGAAGACGAACTGGGTTTCTGAGTTCGATTGCA
                                                  |
                                                 6976
```

**L-segment 190-6936**

```
                                                190
                                                 |
ACTGCTTCAAGAAGGTTTTAAACTTCTCTCCAACCTTGTGGAGTTAGAGTCCTGTGAATCTCATGCATGCCACTTGAACACTTGCCAAAAATATGTTGAT
|    ||    |       ||     | |  ||   |               || |           | | |  |                  |
AAGTTTTTGTCAGCAAACCAACAACATTTGCAATTGTACCTCCCATATAGACTGGGCTCTGCCGTTCTTCTAAATTCTGCAGCTTGTGGTGGCTTCTCGA
                                                  |
                                                 6936
```

**L-segment 460-6348**

```
                                                460
                                                 |
ACAAGACCTTCTGAGGTCAGGCATCACACTTGTTCCTGTTGTTGACGGTAGAACTAATTATTCTAATAGGTTCACTCCTGAATGGGTGGTGGAGAGGATC
||         |                  |   |  |   |  |     |||   | |   |  |  ||   || |   |   |    ||    |
ACTGAGAAAGTTTCTACTTACAGAGTGGAGTTGACTGGTGATATAATAGTGAAGGCAATTGGCGCACTGGACGACCCTGAAGATGTGAATGCTTTGTTGA
                                                  |
                                                 6348
```

**Supplementary Figure S5. Absence of sequence identities for del-DVGs.** For the most common del-DVGs of both the S- and the L-segment 100 nt regions surrounding the break start/stop positions were examined for sequence identity. For each del-DVG, these regions around the break start are shown in blue, and around the break stop in red, with break start/stops highlighted in bold face. Sequence identities are indicated.

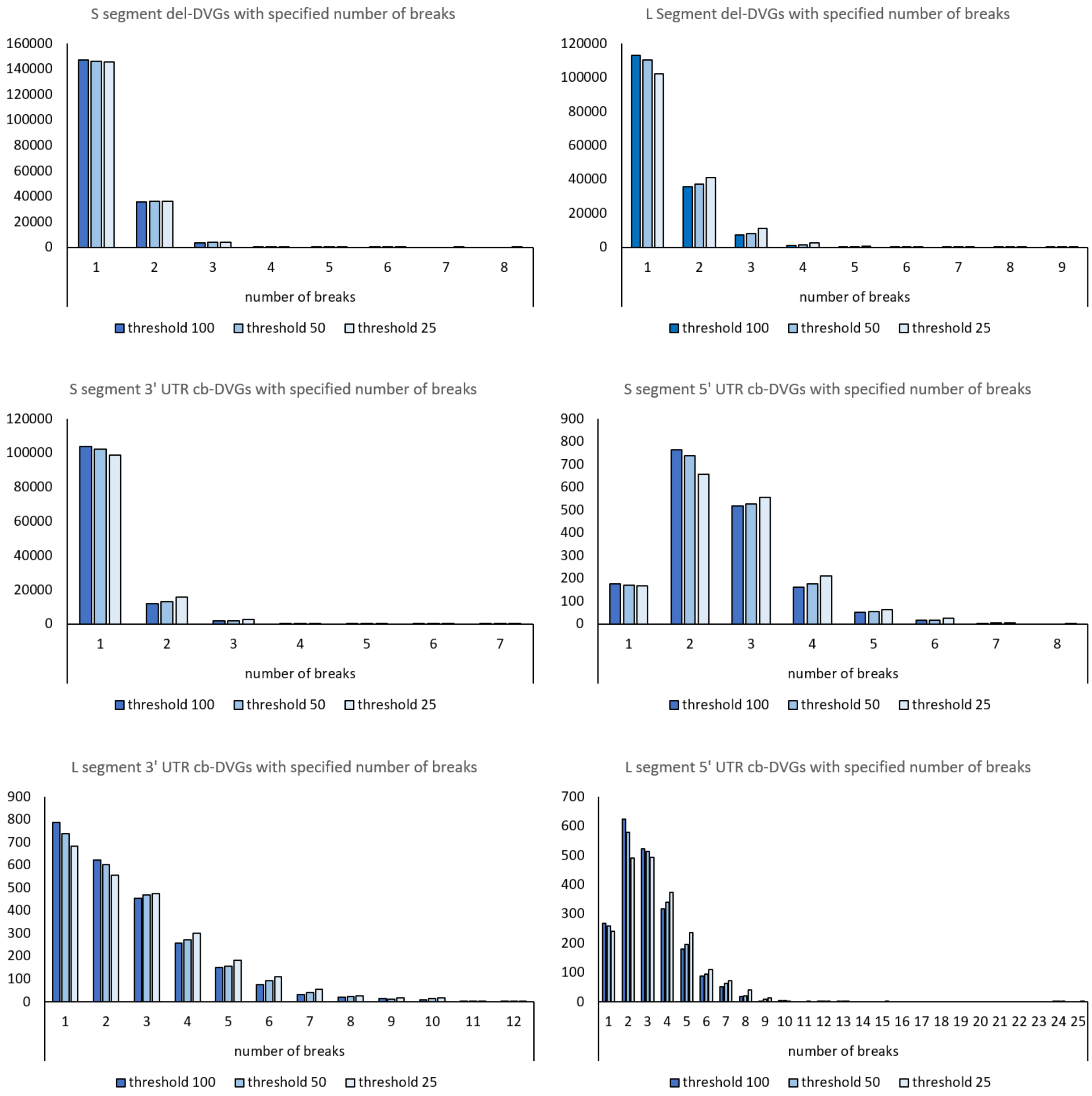


**Supplementary Figure S6. Differences in the number of breaks identified using different thresholds for the minimum gap length to identify a DVG.** The analysis of del-DVGs and cb-DVGs was repeated, lowering the threshold for the minimum gap length required to report a break from 100 nt to 50 nt or 25 nt, as indicated. The number of resulting breaks for the different DVG species is shown.

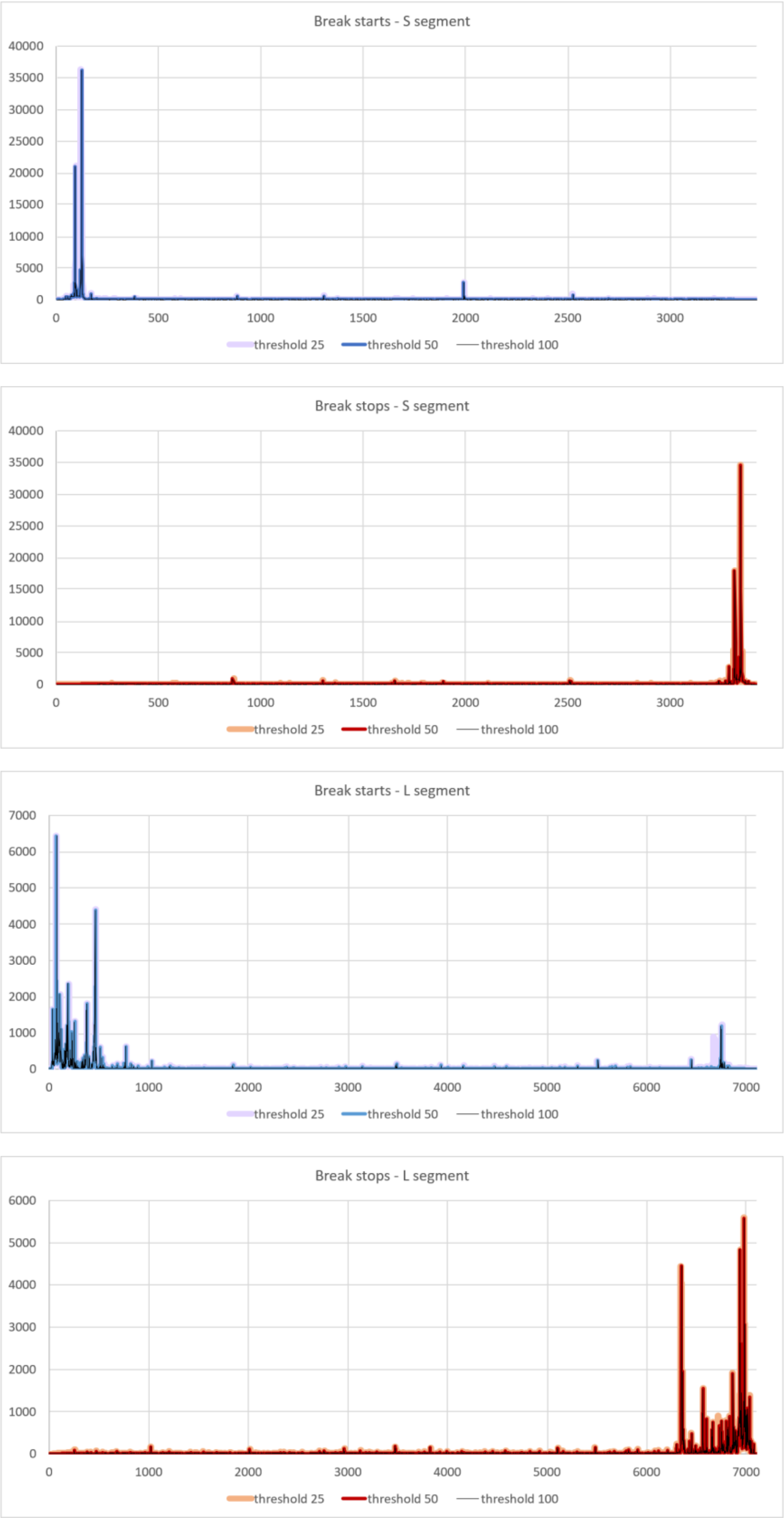


**Supplementary Figure S7. Differences in break starts and stops for del-DVGs when using different thresholds for the minimum gap length to identify a DVG.** The analysis of del-DVGs was repeated, lowering the threshold for the minimal gap length required to report a break from 100 nt to 50 nt or 25 nt. The frequency of identified break starts and stops are shown.

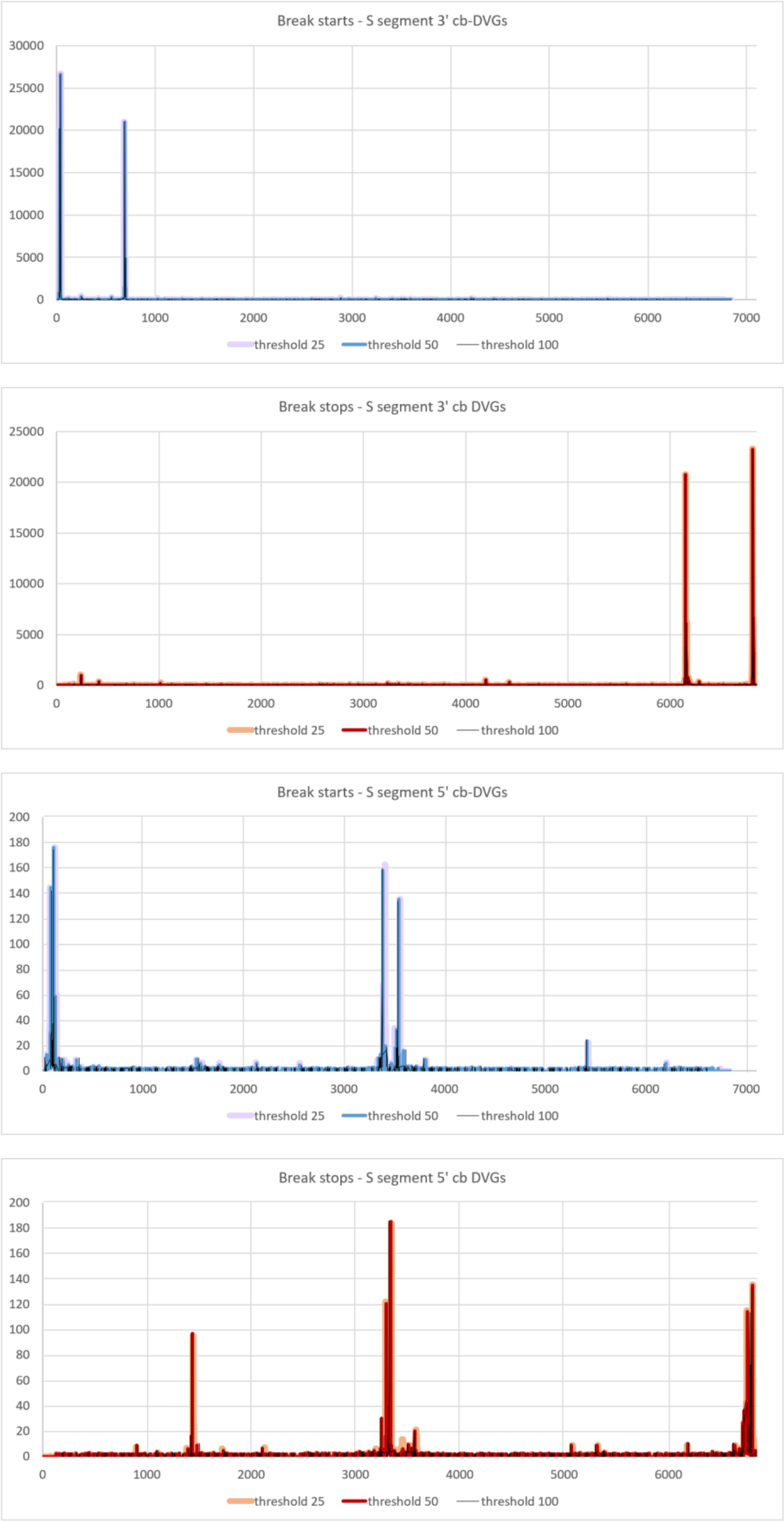


**Supplementary Figure S8. Differences in break starts and stops for S segment cb-DVGs when using different thresholds for the minimum gap length to identify a DVG.** The analysis of cb-DVGs was repeated, lowering the threshold for the minimal gap length required to report a break from 100 nt to 50 nt or 25 nt. The frequency of identified break starts and stops are shown.

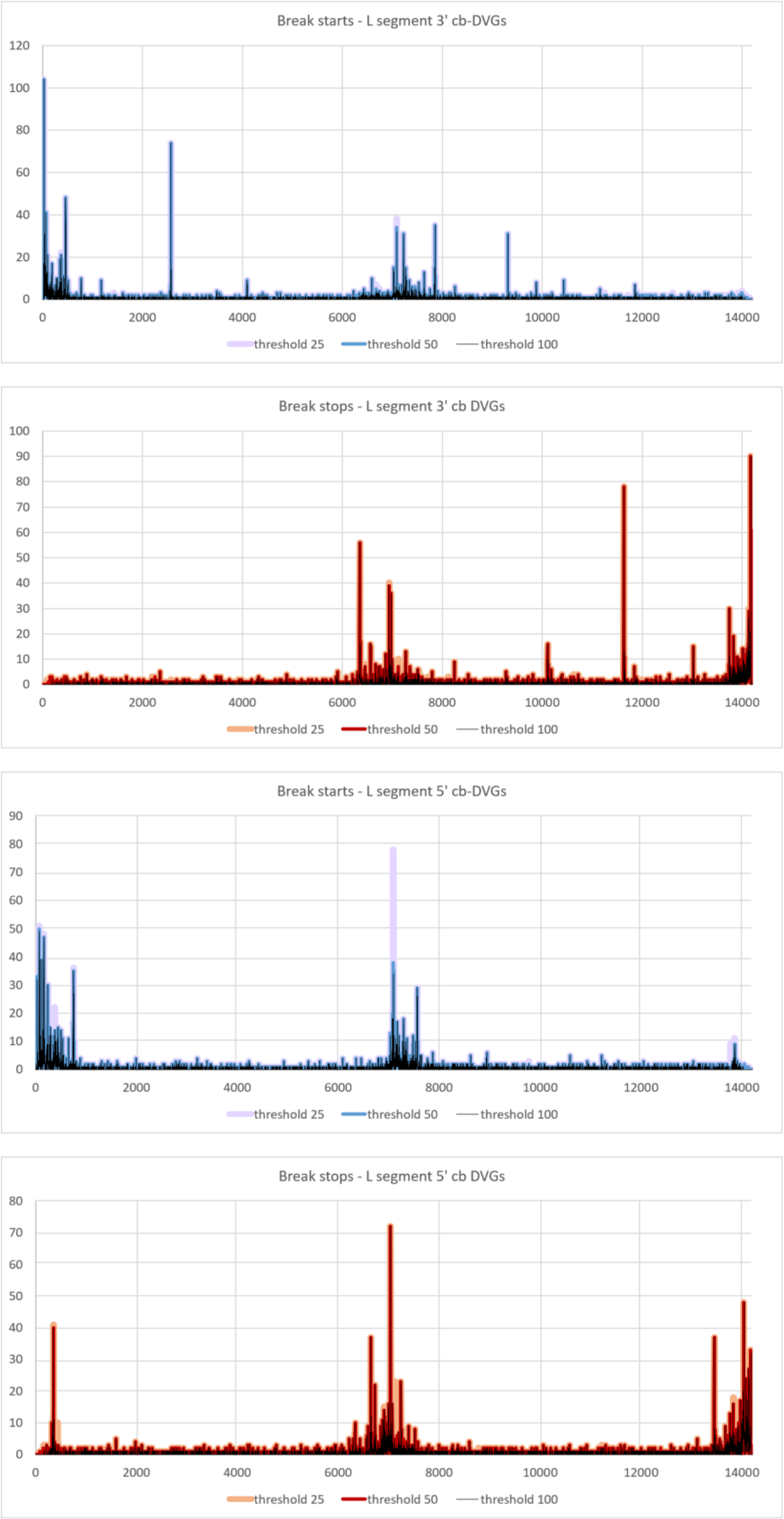


**Supplemental Figure 9. Differences in break starts and stops for L segment cb-DVGs when using different thresholds for the minimum gap length to identify a DVG.** The analysis of cb-DVGs was repeated, lowering the threshold for the minimal gap length required to report a break from 100 nt to 50 nt or 25 nt. The frequency of identified break starts and stops are shown.
