# Supplementary material for: Molecular characterization of arenavirus defective viral genomes reveals sequence features associated with their formation
Source: J Virol. 2025 Dec 9;100(1):e01978-25. doi: 10.1128/jvi.01978-25 (PMC12817923; doi:10.1128/jvi.01978-25)
Supplement: Supplemental text — Scripts used for data analysis. [file jvi.01978-25-s0002.pdf]

analyze_all_DVGs

```
#!/bin/bash
# script analyze_all_DVGs
# analyzes DVGs for all fastq files in current directory
# expects in the current directory 9 fasta files containing barcodes (called TCRV_barcodes.fasta) and vRNA/cRNA/vRNA_cRNA/cRNA_vRNA sequences for S and L segment (called TCRV_S_vRNA.fasta etc.)
# requires flexbar (version 3.1), lastal (version 921), lastdb (version 921), needle (version EMBOSS:6.6.0.0) to be installed

echo "analyze_all_DVGs version 0.21"

for FILENAME in *.fastq; do
   echo
   date +"%T"
   DIRNAME=$(echo $FILENAME | cut -d "." -f 1)
   echo "processing $FILENAME"
   echo "copying files"
   mkdir $DIRNAME
   cd $DIRNAME
   cp ../$FILENAME ./reads.fastq
   cp ../TCRV_barcodes.fasta ./barcodes.fasta
   echo "demultiplexing reads"
   flexbar -r reads.fastq -b barcodes.fasta -bn 200 -bt 0.27 -bk -bg -1
   echo "analyzing S segment reads"
   mkdir S
   cat flexbarOut_barcode_TCRV_S_genome.fastq flexbarOut_barcode_TCRV_S_antigenome.fastq >S/S.fastq
   cd S
   cp ../../TCRV_S_vRNA.fasta .
   cp ../../TCRV_S_cRNA.fasta .
   cp ../../TCRV_S_vRNA_cRNA.fasta .
   cp ../../TCRV_S_cRNA_vRNA.fasta .
   analyze_DVGs TCRV_S_vRNA.fasta TCRV_S_cRNA.fasta TCRV_S_vRNA_cRNA.fasta TCRV_S_cRNA_vRNA.fasta S.fastq
   test -f vRNA.alignments && cat vRNA.alignments >>../../S_vRNA.alignments
   test -f cRNA.alignments && cat cRNA.alignments >>../../S_cRNA.alignments
   test -f vRNA_cRNA.alignments && cat vRNA_cRNA.alignments >>../../S_vRNA_cRNA.alignments
   test -f cRNA_vRNA.alignments && cat cRNA_vRNA.alignments >>../../S_cRNA_vRNA.alignments
   test -f vRNA.breakpoints && cat vRNA.breakpoints >>../../S_vRNA.breakpoints
   test -f cRNA.breakpoints && cat cRNA.breakpoints >>../../S_cRNA.breakpoints
   test -f vRNA_cRNA.breakpoints && cat vRNA_cRNA.breakpoints >>../../S_vRNA_cRNA.breakpoints
   test -f cRNA_vRNA.breakpoints && cat cRNA_vRNA.breakpoints >>../../S_cRNA_vRNA.breakpoints
   cd ..
   echo
   echo "analyzing L segment reads"
   mkdir L
   cat flexbarOut_barcode_TCRV_L_genome.fastq flexbarOut_barcode_TCRV_L_antigenome.fastq >L/L.fastq
   cd L
   cp ../../TCRV_L_vRNA.fasta .
   cp ../../TCRV_L_cRNA.fasta .
   cp ../../TCRV_L_vRNA_cRNA.fasta .
   cp ../../TCRV_L_cRNA_vRNA.fasta .
   analyze_DVGs TCRV_L_vRNA.fasta TCRV_L_cRNA.fasta TCRV_L_vRNA_cRNA.fasta TCRV_L_cRNA_vRNA.fasta L.fastq
   test -f vRNA.alignments && cat vRNA.alignments >>../../L_vRNA.alignments
   test -f cRNA.alignments && cat cRNA.alignments >>../../L_cRNA.alignments
   test -f vRNA_cRNA.alignments && cat vRNA_cRNA.alignments >>../../L_vRNA_cRNA.alignments
   test -f cRNA_vRNA.alignments && cat cRNA_vRNA.alignments >>../../L_cRNA_vRNA.alignments
   test -f vRNA.breakpoints && cat vRNA.breakpoints >>../../L_vRNA.breakpoints
   test -f cRNA.breakpoints && cat cRNA.breakpoints >>../../L_cRNA.breakpoints
   test -f vRNA_cRNA.breakpoints && cat vRNA_cRNA.breakpoints >>../../L_vRNA_cRNA.breakpoints
   test -f cRNA_vRNA.breakpoints && cat cRNA_vRNA.breakpoints >>../../L_cRNA_vRNA.breakpoints
   cd ..
   cd ..
```

```
done
echo
date +"%T"
echo "finished"
```

analyze_DVGs

```
#!/bin/bash
# script analyze_DVGs
# generates alignments and breakpoint files for a given fastq file using reference files for vRNA, cRNA, vRNA_cRNA and cRNA_vRNA
# expects 5 parameters: 4 fasta files with vRNA, cRNA, vRNA_cRNA, and cRNA_vRNA reference, and 1 fastq file with reads
# requires lastal (version 921), lastdb (version 921), needle (version EMBOSS:6.6.0.0) to be installed

if [ "$#" -ne 5 ]; then
    echo
    echo "usage: analyze_DVGs <vRNA_reference_file.fasta> <cRNA_reference_file.fasta> <vRNA_cRNA_reference_file.fasta> <cRNA_vRNA_reference_file.fasta> <read_file.fastq>"
    echo
    exit 0
fi

# assign command line parameters to variables
vRNA_FILE=$1
cRNA_FILE=$2
vRNA_cRNA_FILE=$3
cRNA_vRNA_FILE=$4
READ_FILE=$5

# perform local alignments against possible forms
echo "generating local vRNA alignments"
lastdb vRNA.index $vRNA_FILE
lastal -s 1 -Q 1 vRNA.index $READ_FILE >vRNA.last

echo "generating local cRNA alignments"
lastdb cRNA.index $cRNA_FILE
lastal -s 1 -Q 1 cRNA.index $READ_FILE >cRNA.last

echo "generating local vRNA_cRNA alignments"
lastdb vRNA_cRNA.index $vRNA_cRNA_FILE
lastal -s 1 -Q 1 vRNA_cRNA.index $READ_FILE >vRNA_cRNA.last

echo "generating local cRNA_vRNA alignments"
lastdb cRNA_vRNA.index $cRNA_vRNA_FILE
lastal -s 1 -Q 1 cRNA_vRNA.index $READ_FILE >cRNA_vRNA.last

#convert into sorted position information
echo "extracting position information from local alignments"
cat vRNA.last | convert_last.pl | sort > vRNA.sortedlast
cat cRNA.last | convert_last.pl | sort > cRNA.sortedlast
cat vRNA_cRNA.last | convert_last.pl | sort > vRNA_cRNA.sortedlast
cat cRNA_vRNA.last | convert_last.pl | sort > cRNA_vRNA.sortedlast
sort vRNA.sortedlast cRNA.sortedlast vRNA_cRNA.sortedlast cRNA_vRNA.sortedlast > all.sortedlast

# analyze to determine which DVG type is present
echo "determining DVG type"
cat all.sortedlast | analyze_sorted_last.pl > all.analyzed
mkdir vRNA
mkdir cRNA
mkdir vRNA_cRNA
mkdir cRNA_vRNA
grep "^vRNA " all.analyzed > vRNA/vRNA.analyzed
grep "^cRNA " all.analyzed > cRNA/cRNA.analyzed
grep "^vRNA_cRNA " all.analyzed > vRNA_cRNA/vRNA_cRNA.analyzed
grep "^cRNA_vRNA " all.analyzed > cRNA_vRNA/cRNA_vRNA.analyzed

# crop reads and reference sequences
echo "running global alignments for vRNA"
echo "cropping sequences"
cd vRNA
cat vRNA.analyzed | generate_cropped_sequences.pl ../$vRNA_FILE ../$READ_FILE
echo "aligning sequences"
needle_DVGs
echo "running global alignments for cRNA"
echo "cropping sequences"
cd ../cRNA
```

```
68  cat cRNA.analyzed | generate_cropped_sequences.pl ../$cRNA_FILE ../$READ_FILE
69  echo "aligning sequences"
70  needle_DVGs
71  echo "running global alignments for vRNA_cRNA"
72  echo "cropping sequences"
73  cd ../vRNA_cRNA
74  cat vRNA_cRNA.analyzed | generate_cropped_sequences.pl ../$vRNA_cRNA_FILE ../
    $READ_FILE
75  echo "aligning sequences"
76  needle_DVGs
77  echo "running global alignments for cRNA_vRNA"
78  echo "cropping sequences"
79  cd ../cRNA_vRNA
80  cat cRNA_vRNA.analyzed | generate_cropped_sequences.pl ../$cRNA_vRNA_FILE ../
    $READ_FILE
81  echo "aligning sequences"
82  needle_DVGs
83  cd ..
84
85  # analyze output data
86  echo "compressing needle results - output can be found in xxx.alignments"
87  test -f vRNA/alignments.needle && cat vRNA/alignments.needle |
    compress_needle_output.pl > vRNA.alignments
88  test -f cRNA/alignments.needle && cat cRNA/alignments.needle |
    compress_needle_output.pl > cRNA.alignments
89  test -f vRNA_cRNA/alignments.needle && cat vRNA_cRNA/alignments.needle |
    compress_needle_output.pl > vRNA_cRNA.alignments
90  test -f cRNA_vRNA/alignments.needle && cat cRNA_vRNA/alignments.needle |
    compress_needle_output.pl > cRNA_vRNA.alignments
91
92  echo "finding breakpoints - output can be found in xxx.breakpoints"
93  test -f vRNA/alignments.needle && cat vRNA/alignments.needle |
    analyze_needle_output.pl > vRNA.breakpoints
94  test -f cRNA/alignments.needle && cat cRNA/alignments.needle |
    analyze_needle_output.pl > cRNA.breakpoints
95  test -f vRNA_cRNA/alignments.needle && cat vRNA_cRNA/alignments.needle |
    analyze_needle_output.pl > vRNA_cRNA.breakpoints
96  test -f cRNA_vRNA/alignments.needle && cat cRNA_vRNA/alignments.needle |
    analyze_needle_output.pl > cRNA_vRNA.breakpoints
```

needle_DVGs

```
#!/bin/bash
# script needle_DVGs
# creates global alignments of 2 fasta files (called read.fasta and reference.fasta) in all subdirectories
# requires needle (version EMBOSS:6.6.0.0) to be installed

for d in */; do
    if [ -d "$d" ]; then
        cd $d
        needle reference.fasta read.fasta -gapopen 50 -gapextend 0.000000001 -endweight Y -endopen 5 -endextend 0.000000001 -outfile alignment.needle >/dev/null 2>/dev/null
        cat alignment.needle >> ../alignments.needle
        cd ..
    fi
done

```

convert_last.pl

```perl
#!/usr/bin/perl

# reformats last-output (format -j 3) during DVG analysis
# expects input from <STDIN>
# output format: <query name> <reference name> <reference start> <reference length> <query start> <query length> <score>
# positions are given with 5 digits, adding leading zeros if necessary, to allow sorting by piping to linux command sort
# example usage: cat vRNA.last | convertlast.pl | sort > vRNA.sortedlast

use warnings;
use strict;

my $firstline;
my $secondline;
my $thirdline;
my $fourthline;
my @firstline;
my @secondline;
my @thirdline;
my @fourthline;
my $queryname;
my $querystart;
my $querylength;
my $referencename;
my $referencestart;
my $referencelength;
my $score;

# get next match from last file
sub getnextmatch
{
    do {$firstline = <>; exit unless defined $firstline} until $firstline =~ /^a/;
    $secondline = <>;
    $thirdline = <>;
    $fourthline = <>;
    @firstline = split (" ",$firstline);
    @secondline = split (" ",$secondline);
    @thirdline = split (" ",$thirdline);
    @fourthline = split (" ",$fourthline);
    $queryname = $thirdline[1];
    $referencename = $secondline[1];
    $referencestart = $secondline[2];
    $referencelength = $secondline[3];
    $querystart = $thirdline[2];
    $querylength = $thirdline[3];
    $score = $firstline[1];
}

while (1)
{
    &getnextmatch;
    print "$queryname $referencename ";
    print (sprintf  ("%05d %05d %05d %05d", $referencestart, $referencelength, $querystart, $querylength));     # sprintf adds leading zeros if necessary
    print (" $score\n");
}

```

generate_cropped_sequences.pl

```perl
#!/usr/bin/perl

# generates cropped sequences for DVG analysis
# expects 2 input files: generate_cropped_sequences.pl <reference.fasta> <queries.fastq>
# the first file is the reference sequence in fasta format
# the second file is a file with all reads in fastq format
# also expects mapping information from STDIN - this is usually the output from analyze_sorted_last.pl grepped for one kind of DVG-type

use warnings;
use strict;
use List::Util qw(max);

my $reference_sequence="";
my %read_sequence;
my $filler_sequence="N" x 10000;
my $read_output_filehandle;
my $reference_output_filehandle;

# loads reference sequence into $reference_sequence
# expects filename for a fasta file as parameter
sub loadreference
{
    my ($reference_filename) = @_;
    open (my $reference_filehandle, "<", $reference_filename) or die "Can't open $reference_filename\n";
    while (my $reference_fileline = <$reference_filehandle>)
    {
        $reference_fileline =~ s/\R//g; # removes both linux (<NL>) and windows (<NL><CR>) line breaks
        $reference_sequence=$reference_sequence.$reference_fileline unless $reference_fileline =~ /^>/;
    }
    close ($reference_filehandle);
    #print STDERR "loaded reference sequence $reference_filename:\n$reference_sequence\n";
    #print STDERR length($reference_sequence)." characters loaded\n";
    print STDERR "loaded reference sequence $reference_filename\n";
}

# load sequence reads
# expectes filename for a fastq file as a parameter
# loads fastq file only based on line numbers, expexts in first (fifth, ninth etc.) line read name in format "@<name> " and in second (sixth etc.) line sequence data
sub loadreads
{
    my ($read_filename) = @_;
    my $number_of_reads = 0;
    open (my $read_filehandle, "<", $read_filename) or die "Can't open $read_filename\n";
    while (my $read_firstline = <$read_filehandle>)
    {
        my $read_secondline = <$read_filehandle>;
        my $read_thirdline = <$read_filehandle>;
        my $read_fourthline = <$read_filehandle>;
        my @read_firstline = split (" ",$read_firstline);
        my $read_name = substr $read_firstline[0],1;
        chomp $read_secondline;
        $read_sequence{$read_name}=$read_secondline;
        $number_of_reads++;
    }
    close ($read_filehandle);
    print STDERR "loaded $number_of_reads reads from $read_filename:\n";
}

# generates subdir and two filehandles for reference file and read file
# expects directory name as parameter
# filehandles are put into global variables $read_output_filehandle and $reference_output_filehandle
# terminates program if directory or any of the
sub prepareoutput
```

```
64  {
65      my ($dir_name) = @_;
66      mkdir $dir_name;
67      open ($read_output_filehandle,">", $dir_name."/read.fasta") or die ("Can't create
        $dir_name"."/read.fasta");
68      open ($reference_output_filehandle,">", $dir_name."/reference.fasta") or die (
        "Can't create $dir_name"."/reference.fasta");
69  }
70
71  # MAIN PROGRAM
72
73  # first ensure program was called correctly
74  die "usage: generate_cropped_sequences.pl <reference.fasta> <queries.fastq>\nexpects
    cropping information as provided by analyze_sorted_last.pl from <STDIN>\n" unless (
    $#ARGV == 1);
75
76  # define variables
77  my $nextline;
78  my @nextline;
79  my $number_of_cropped_reads = 0;
80
81  # load files
82  &loadreference($ARGV[0]);
83  &loadreads($ARGV[1]);
84
85  $nextline = <STDIN>;
86
87  while (defined($nextline))
88  {
89      @nextline = split(" ",$nextline);
90      my $number_of_fields = @nextline;
91      my $read_name = $nextline[1];
92      my $reference_start = $nextline[2];
93      my $reference_stop = $nextline[$number_of_fields-5]+$nextline[$number_of_fields-4
        ];
94      my $read_start = $nextline[4];
95      my $read_stop = $nextline[$number_of_fields-3]+$nextline[$number_of_fields-2];
96      my $formatted_reference_start = sprintf("%05d", $reference_start+1);
97      my $formatted_reference_stop = sprintf("%05d", $reference_stop+1);
98      my $formatted_read_start = sprintf("%05d", $read_start+1);
99      my $formatted_read_stop = sprintf("%05d", $read_stop+1);
100     my $reference_length = $reference_stop-$reference_start;
101     my $cropped_reference = substr($reference_sequence, $reference_start,
        $reference_length);
102     my $cropped_read_length = $read_stop-$read_start;
103     my $cropped_read = substr($read_sequence{$read_name}, $read_start,
        $cropped_read_length);
104     &prepareoutput($read_name);
105     print $reference_output_filehandle ">REF_"."$formatted_reference_start"."_to_"."
        $formatted_reference_stop"."\n$cropped_reference\n\n";
106     print $read_output_filehandle ">$read_name"."_cropped_"."$formatted_read_start".
        "_to_"."$formatted_read_stop"."\n$cropped_read\n\n";
107     $number_of_cropped_reads++;
108     $nextline = <STDIN>;
109 }
110 print STDERR "processed $number_of_cropped_reads reads\n";
```

compress_needle_output.pl

```perl
#!/usr/bin/perl

# compresses needle output file into a more readable format

use warnings;
use strict;
use List::Util qw(max);

# define variables
my $nextline;
my $firstline;
my $secondline;
my $thirdline;
my $currentread;
my @thirdline;
$currentread = "";

$nextline = <STDIN>;

while (defined($nextline))
{
    if ((substr($nextline,0,1) ne "#") && ($nextline ne "\n"))
    {
        $firstline = $nextline;
        $secondline = <STDIN>;
        $thirdline = <STDIN>;
        @thirdline = split(" ", $thirdline);
        if ($thirdline[0] ne $currentread)
        {
            $currentread = $thirdline[0];
            print
            "################################################################################
            #####\n\n";
        }
        if ($secondline =~ /\S/) # match any non white space
        {
            print $firstline;
            print $secondline;
            print $thirdline;
            print "\n";
        }
    }
    $nextline = <STDIN>;
}
```

analyze_needle_output.pl

```perl
#!/usr/bin/perl

# analyzes needle output file for breakpoints
# format for output: <read_name> <breakpoint start 1> <breakpoint stop 1> [<breakpoint start n> <breakpoint stop n>]
# breakpoint start is the last position which still aligns (starting at +1), breakpoint stop is the first position that aligns again

use warnings;
use strict;
use List::Util qw(max);

# define variables
my @readname;
my @readseq;
my @alignment;
my @refseq;
my @breakpoints;
my @refoffset;
my $numberofreads = 0;

# constant to define cutoff for breakpoints, if gap is longer than $breakinggap, it is interpreted as a break
my $breakinggap=100;

# loads all alignments into arrays @readname, @readseq, @alignment, @refseq, and @refoffset; arrays start at 0; loads number of reads into $numberofreads
sub getalignments
{
    my $nextline;
    my $firstline;
    my $secondline;
    my $thirdline;
    my $currentreadname = "";
    my $readindex = -1;
    my @firstline;
    my @thirdline;
    $nextline = <STDIN>;
    while (defined($nextline))
    {
        if ((substr($nextline,0,1) ne "#") && ($nextline ne "\n"))
        {
            $firstline = $nextline;
            $secondline = <STDIN>;
            $thirdline = <STDIN>;
            @firstline = split(" ", $firstline);
            $secondline = substr($secondline, 21, 50);
            @thirdline = split(" ", $thirdline);
            if ($thirdline[0] ne $currentreadname)
            {
                $currentreadname = $thirdline[0];
                $readindex ++;
                $numberofreads = $readindex+1;
                $readname[$readindex] = $currentreadname;
                $readseq[$readindex] = "";
                $alignment[$readindex] = "";
                $refseq[$readindex] = "";
                $refoffset[$readindex] = substr($firstline, 4, 5)-1;
            }
            $refseq[$readindex] = $refseq[$readindex].$firstline[2];
            $alignment[$readindex] = $alignment[$readindex].$secondline;
            $readseq[$readindex] = $readseq[$readindex].$thirdline[2];
        }
        $nextline = <STDIN>;
    }
}

# determines breakpoints in alignments and writes the results into array @breakpoints
# format for $breakpoint[]: <read_name> <breakpoint start 1> <breakpoint stop 1> [<breakpoint start n> <breakpoint stop n>]
# breakpoint start is the last position which still aligns (starting at +1) breakpoint stop is the first position that aligns again
```

```
67  sub analyzealignments
68  {
69      my $readindex;
70      my $gap;
71      my $nongap;
72      my $position;
73      my $breakpointstart;
74      my $breakpointstop;
75      my $breakpointlength;
76      my $currentalignment;
77      my $currentreference;
78      my $reference_until_break_start;
79      my $reference_until_break_stop;
80      my $number_of_gaps_in_ref_until_start;
81      my $number_of_gaps_in_ref_until_stop;
82      for ($readindex = 0; $readindex < $numberofreads; $readindex++)
83      {
84          $breakpoints[$readindex]=$readname[$readindex];
85          $currentalignment = $alignment[$readindex];
86          $currentreference = $refseq[$readindex];
87          $position = $refoffset[$readindex];
88          while ($currentalignment ne "")
89          {
90              if (($nongap , $gap) = $currentalignment =~ /([\.\|]+)( *)/)    # extract
                next nongap and gap using a regular expression
91              {
92                  $breakpointstart = $position + length($nongap);
93                  $breakpointstop = $position + length($nongap)+length($gap)+1;
94                  $reference_until_break_start = substr($currentreference, 0,
                    $breakpointstart-$refoffset[$readindex]);
95                  $reference_until_break_stop = substr($currentreference, 0,
                    $breakpointstop-$refoffset[$readindex]-1);
96                  $number_of_gaps_in_ref_until_start = () = $reference_until_break_start
                     =~ /\-/gi;   # compensate for gaps in reference sequence
97                  $number_of_gaps_in_ref_until_stop = () = $reference_until_break_stop
                    =~ /\-/gi; # compensate for gaps in reference sequence
98                  $breakpointlength = ($breakpointstop-$number_of_gaps_in_ref_until_stop
                    )-($breakpointstart-$number_of_gaps_in_ref_until_start)-1;
99                  if ($breakpointlength >= $breakinggap)
100                 {
101                     $breakpoints[$readindex] = $breakpoints[$readindex]." ".(
                        $breakpointstart-$number_of_gaps_in_ref_until_start)." ".(
                        $breakpointstop-$number_of_gaps_in_ref_until_stop);
102                 }
103                 $currentalignment = substr($currentalignment, length($nongap.$gap),
                    length($currentalignment)-length($nongap.$gap));
104                 $position = $position + length($nongap.$gap);
105             } else
106             {
107                 $currentalignment="";
108             }
109         }
110         $breakpoints[$readindex]=$breakpoints[$readindex]."\n";
111     }
112 }
113
114 &getalignments;
115 &analyzealignments;
116 for (my $readindex = 0; $readindex < $numberofreads; $readindex++)
117 {
118     print $breakpoints[$readindex];
119 }
120
121
```

analyze_sorted_last.pl

```perl
#!/usr/bin/perl

# analyzes sorted outputs from 4 different convertedlast runs (for vRNA, cRNA, vRNA_cRNA, and cRNA_vRNA during DVG analysis
# expects input from <STDIN>
# output format <DVG-type> <query name> [<reference position> <reference length> <query position> <query length> score=<score>]
# DVG-type can be "vRNA", "cRNA", "vRNA_cRNA", "cRNA_vRNA", "single_seq_only", or "unsure_about"

use constant S_vRNA_ID => "TCRV_S_vRNA";
use constant S_cRNA_ID => "TCRV_S_cRNA";
use constant S_vRNA_cRNA_ID => "TCRV_S_vRNA_cRNA";
use constant S_cRNA_vRNA_ID => "TCRV_S_cRNA_vRNA";
use constant L_vRNA_ID => "TCRV_L_vRNA";
use constant L_cRNA_ID => "TCRV_L_cRNA";
use constant L_vRNA_cRNA_ID => "TCRV_L_vRNA_cRNA";
use constant L_cRNA_vRNA_ID => "TCRV_L_cRNA_vRNA";

use warnings;
use strict;
use List::Util qw(max);

my $nextline;
my @nextline;

my $currentqueryname;
my @currentquery_vRNA;
my @currentquery_cRNA;
my @currentquery_vRNA_cRNA;
my @currentquery_cRNA_vRNA;
my $vRNAcount;
my $cRNAcount;
my $vRNA_cRNAcount;
my $cRNA_vRNAcount;

# get next query data from sorted last file
sub get_next_query
{
        @nextline = split (" ",$nextline);

                # initialize variables
        $currentqueryname = $nextline[0];
        @currentquery_vRNA = ();
        @currentquery_cRNA = ();
        @currentquery_vRNA_cRNA = ();
        @currentquery_cRNA_vRNA = ();
        $vRNAcount = 0;
        $cRNAcount = 0;
        $vRNA_cRNAcount = 0;
        $cRNA_vRNAcount = 0;
        while ($nextline[0] eq $currentqueryname)                                                                       # fill appropriate array until new query is reached
        {
                if ($nextline[1] eq S_vRNA_ID || $nextline[1] eq L_vRNA_ID) {$vRNAcount++; $currentquery_vRNA[$vRNAcount] = $nextline;};
                if ($nextline[1] eq S_cRNA_ID || $nextline[1] eq L_cRNA_ID) {$cRNAcount++; $currentquery_cRNA[$cRNAcount] = $nextline};
                if ($nextline[1] eq S_vRNA_cRNA_ID || $nextline[1] eq L_vRNA_cRNA_ID) {$vRNA_cRNAcount ++; $currentquery_vRNA_cRNA[$vRNA_cRNAcount] = $nextline};
                if ($nextline[1] eq S_cRNA_vRNA_ID || $nextline[1] eq L_cRNA_vRNA_ID) {$cRNA_vRNAcount ++; $currentquery_cRNA_vRNA[$cRNA_vRNAcount] = $nextline};
                $nextline = <>; return unless defined($nextline);                                                       # get next line loaded, return if end of file is reached
                @nextline = split(" ",$nextline);
        }
}

```

```
58  # removes double matches, e.g. if query 100 to 200 matches to reference 400 to 500,
    and query 140 to 170 matches to reference 440 to 470.
59  sub remove_double_matches
60  {
61          if ($vRNAcount > 1)
62          {
63                  for (my $i=1; $i < $vRNAcount; $i++)
64                  {
65                          my @query = split(" ",$currentquery_vRNA[$i]);
66                          my @nextquery = split(" ",$currentquery_vRNA[$i+1]);
67                          if ($nextquery[2] < ($query[2]+$query[3]))
                                                                                # if
                            next match starts within the current match (judging by the
                            reference sequence,
68                          {
69                                  my @score = split("=",$query[6]);

                                                # need to split because $query[6] has
                                    the format "score=xxx"
70                                  my @nextscore = split("=",$nextquery[6]);
71                                  if ($score[1]>=$nextscore[1])
72                                  {
73                                          my $j = $i+1;
74                                          while ($j<$vRNAcount)
75                                          {
76                                                  $currentquery_vRNA[$j]=
                                                    $currentquery_vRNA[$j+1];
                                                                # copy all matches in
                                                    array starting with the item after
                                                    the next match one element up
77                                                  $j++;
78                                          }
79                                          $currentquery_vRNA[$j]="";

                                                                # delete now
                                            duplicated match at the end of array
80                                          $vRNAcount--;
81                                  }
82                                  else
83                                  {
84                                          my $j = $i;
85                                          while ($j<$vRNAcount)
86                                          {
87                                                  $currentquery_vRNA[$j]=
                                                    $currentquery_vRNA[$j+1];
                                                                # copy all matches in
                                                    array starting with the item after
                                                    the current match one element up
88                                                  $j++;
89                                          }
90                                          $currentquery_vRNA[$j]="";

                                                                # delete now
                                            duplicated match at the end of array
91                                          $vRNAcount--;
92                                  }
93                          $i--;


                                    # go one step back in case more than 2 matches overlap
94                          }
95
96                  }
97          }
98          if ($cRNAcount > 1)
99          {
100                 for (my $i=1; $i < $cRNAcount; $i++)
101                 {
102                         my @query = split(" ",$currentquery_cRNA[$i]);
103                         my @nextquery = split(" ",$currentquery_cRNA[$i+1]);
104                         if ($nextquery[2] < ($query[2]+$query[3]))
                                                                                # if
```

```
                        next match starts within the current match (judging by the
                        reference sequence,
105                     {
106                             my @score = split("=",$query[6]);

                                        # need to split because $query[6] has
                                the format "score=xxx"
107                             my @nextscore = split("=",$nextquery[6]);
108                             if ($score[1]>=$nextscore[1])
109                             {
110                                     my $j = $i+1;
111                                     while ($j<$cRNAcount)
112                                     {
113                                             $currentquery_cRNA[$j]=
                                                $currentquery_cRNA[$j+1];
                                                        # copy all matches in
                                                array starting with the item after
                                                the next match one element up
114                                             $j++;
115                                     }
116                                     $currentquery_cRNA[$j]="";

                                                        # delete now
                                        duplicated match at the end of array
117                                     $cRNAcount--;
118                             }
119                             else
120                             {
121                                     my $j = $i;
122                                     while ($j<$cRNAcount)
123                                     {
124                                             $currentquery_cRNA[$j]=
                                                $currentquery_cRNA[$j+1];
                                                        # copy all matches in
                                                array starting with the item after
                                                the current match one element up
125                                             $j++;
126                                     }
127                                     $currentquery_cRNA[$j]="";

                                                        # delete now
                                        duplicated match at the end of array
128                                     $cRNAcount--;
129                             }
130                     $i--;


                                # go one step back in case more than 2 matches overlap
131                     }
132
133             }
134     }
135     if ($vRNA_cRNAcount > 1)
136     {
137             for (my $i=1; $i < $vRNA_cRNAcount; $i++)
138             {
139                     my @query = split(" ",$currentquery_vRNA_cRNA[$i]);
140                     my @nextquery = split(" ",$currentquery_vRNA_cRNA[$i+1]);
141                     if ($nextquery[2] < ($query[2]+$query[3]))
                                                                        # if
                        next match starts within the current match (judging by the
                        reference sequence,
142                     {
143                             my @score = split("=",$query[6]);

                                        # need to split because $query[6] has
                                the format "score=xxx"
144                             my @nextscore = split("=",$nextquery[6]);
145                             if ($score[1]>=$nextscore[1])
146                             {
147                                     my $j = $i+1;
148                                     while ($j<$vRNA_cRNAcount)
```

```
149                                     {
150                                             $currentquery_vRNA_cRNA[$j]=
                                                $currentquery_vRNA_cRNA[$j+1];
                                                        # copy all matches in
                                                array starting with the item after
                                                the next match one element up
151                                             $j++;
152                                     }
153                                     $currentquery_vRNA_cRNA[$j]="";

                                                        # delete now duplicated match
                                                at the end of array
154                                     $vRNA_cRNAcount--;
155                             }
156                             else
157                             {
158                                     my $j = $i;
159                                     while ($j<$vRNA_cRNAcount)
160                                     {
161                                             $currentquery_vRNA_cRNA[$j]=
                                                $currentquery_vRNA_cRNA[$j+1];
                                                        # copy all matches in
                                                array starting with the item after
                                                the current match one element up
162                                             $j++;
163                                     }
164                                     $currentquery_vRNA_cRNA[$j]="";

                                                        # delete now duplicated match
                                                at the end of array
165                                     $vRNA_cRNAcount--;
166                             }
167                     $i--;


                                # go one step back in case more than 2 matches overlap
168                     }
169
170             }
171     }
172     if ($cRNA_vRNAcount > 1)
173     {
174             for (my $i=1; $i < $cRNA_vRNAcount; $i++)
175             {
176                     my @query = split(" ",$currentquery_cRNA_vRNA[$i]);
177                     my @nextquery = split(" ",$currentquery_cRNA_vRNA[$i+1]);
178                     if ($nextquery[2] < ($query[2]+$query[3]))
                                                                        # if
                     next match starts within the current match (judging by the
                     reference sequence,
179                     {
180                             my @score = split("=",$query[6]);

                                        # need to split because $query[6] has
                             the format "score=xxx"
181                             my @nextscore = split("=",$nextquery[6]);
182                             if ($score[1]>=$nextscore[1])
183                             {
184                                     my $j = $i+1;
185                                     while ($j<$cRNA_vRNAcount)
186                                     {
187                                             $currentquery_cRNA_vRNA[$j]=
                                                $currentquery_cRNA_vRNA[$j+1];
                                                        # copy all matches in
                                                array starting with the item after
                                                the next match one element up
188                                             $j++;
189                                     }
190                                     $currentquery_cRNA_vRNA[$j]="";

                                                        # delete now duplicated match
                                                at the end of array
```

```
191                                     $cRNA_vRNAcount--;
192                             }
193                             else
194                             {
195                                     my $j = $i;
196                                     while ($j<$cRNA_vRNAcount)
197                                     {
198                                             $currentquery_cRNA_vRNA[$j]=
                                                $currentquery_cRNA_vRNA[$j+1];
                                                        # copy all matches in
                                                array starting with the item after
                                                the current match one element up
199                                             $j++;
200                                     }
201                                     $currentquery_cRNA_vRNA[$j]="";

                                                # delete now duplicated match
                                        at the end of array
202                                     $cRNA_vRNAcount--;
203                             }
204                     $i--;

                                # go one step back in case more than 2 matches overlap
205                     }
206
207             }
208     }
209 }
210
211 # analyzes next query, following these rules:
212 # if there is just one match, return "single match only"
213 # if the vRNA/cRNA count is as high or higher than all other counts, it has to be a
    vRNA/cRNA internal deletion
214 # vRNA_cRNA/cRNA_vRNA count is highest, it should be a copy-back DVG, but only in one
    of the two will the order of matches be possible
215 sub analyze_query
216 {
217         my $maxcount = max($vRNAcount, $cRNAcount, $vRNA_cRNAcount, $cRNA_vRNAcount);
218         if ($maxcount == 1)
219         {
220                 print "single_seq_only $currentqueryname\n";
221                 return;
222         }
223         if ($vRNAcount == $maxcount)
224         {
225                 print "vRNA $currentqueryname";
226                 for (my $i=1; $i <= $vRNAcount; $i++)
227                 {
228                         my @query = split(" ",$currentquery_vRNA[$i]);
229                         print(" $query[2] $query[3] $query[4] $query[5] $query[6]");
230                 }
231                 print "\n";
232                 return;
233         }
234         if ($cRNAcount == $maxcount)
235         {
236                 print "cRNA $currentqueryname";
237                 for (my $i=1; $i <= $cRNAcount; $i++)
238                 {
239                         my @query = split(" ",$currentquery_cRNA[$i]);
240                         print(" $query[2] $query[3] $query[4] $query[5] $query[6]");
241                 }
242                 print "\n";
243                 return;
244         }
245         if ($vRNA_cRNAcount == $maxcount)
246         {
247                 my $could_be_vRNA_cRNA = 1;
248                 for (my $i=1; $i < $vRNA_cRNAcount; $i++)
249                 {
250                         my @query = split(" ",$currentquery_vRNA_cRNA[$i]);
```

```
251                         my @nextquery = split (" ",$currentquery_vRNA_cRNA[$i+1]);
252                         if ($query[4] >= $nextquery[4]) {$could_be_vRNA_cRNA = 0}
                                                # compare query positions; if they are
                            not in order, this query can't be vRNA_cRNA; NOTE: reference
                            positions are in order due to sorting - if this is not the
                            case, the program won't work
253                         if ($i == $vRNA_cRNAcount-1 && $could_be_vRNA_cRNA == 1)
                                                 # if all query positions have been
                            tested and are in order, then it is vRNA_cRNA
254                         {
255                                 print "vRNA_cRNA $currentqueryname";
256                                 for (my $j=1; $j <= $vRNA_cRNAcount; $j++)
257                                 {
258                                         my @query = split(" ",$currentquery_vRNA_cRNA[
                                            $j]);
259                                         print(" $query[2] $query[3] $query[4] $query
                                            [5] $query[6]");
260                                 }
261                                 print "\n";
262                                 return;
263                         }
264                 }
265         }
266         if ($cRNA_vRNAcount == $maxcount)
267         {
268                 my $could_be_cRNA_vRNA = 1;
269                 for (my $i=1; $i < $cRNA_vRNAcount; $i++)
270                 {
271                         my @query = split(" ",$currentquery_cRNA_vRNA[$i]);
272                         my @nextquery = split (" ",$currentquery_cRNA_vRNA[$i+1]);
273                         if ($query[4] >= $nextquery[4]) {$could_be_cRNA_vRNA = 0}
                                                # compare query positions; if they are
                            not in order, abort; NOTE: reference positions are in order
                            due to sorting - if this is not the case, the program won't
                            work
274                         if ($i == $cRNA_vRNAcount-1 && $could_be_cRNA_vRNA == 1)
                                                 # if all query positions have been
                            tested and are in order, then it is cRNA_vRNA
275                         {
276                                 print "cRNA_vRNA $currentqueryname";
277                                 for (my $j=1; $j <= $cRNA_vRNAcount; $j++)
278                                 {
279                                         my @query = split(" ",$currentquery_cRNA_vRNA[
                                            $j]);
280                                         print(" $query[2] $query[3] $query[4] $query
                                            [5] $query[6]");
281                                 }
282                                 print "\n";
283                                 return;
284                         }
285                 }
286         }
287         print "unsure_about $currentqueryname\n";
288 }
289
290 # MAIN PROGRAM
291
292 $nextline = <>;
                                                 # get first line loaded, exits if
    file is empty
293
294 while (defined($nextline))
295 {
296     &get_next_query;
297     &remove_double_matches;
298         &analyze_query;
299 }
300
```
